# Complex function solution for deformation and failure mechanism of inclined coal seam roadway

**DOI:** 10.1038/s41598-022-11277-7

**Published:** 2022-05-03

**Authors:** Xianyu Xiong, Jun Dai, Chen Xinnian, Yibo Ouyang

**Affiliations:** 1 School of Architecture and Civil Engineering, Xi’an University of Science and Technology, Yan’ta Middle Road No.58, Xi’an, 710054 Shaanxi China; 2 College of Energy Science and Engineering, Xi’an University of Science and Technology, Xi’an, 710054 Shaanxi China

**Keywords:** Civil engineering, Energy infrastructure

## Abstract

The stressed environment of the inclined coal seam roadway is complex and changeable, and the damage degree of surrounding rock increases, threatening the safe mining of coal mines. In order to take targeted support measures to control the stability of roadway surrounding rock, it is very important to study the stress and deformation characteristics of roadway surrounding rock in inclined coal seam. Therefore, this paper analyzes the deformation and failure law of inclined coal seam roadway according to the theory of complex variable function. It optimizes the solution process and accuracy of the mapping function coefficient and deduces the analytical solution of surrounding rock stress and deformation inclined coal seam roadway. The deformation and failure mechanism of surrounding rock in inclined coal seam roadway is revealed theoretically, and further use numerical simulation and physical simulation tests for supplementary analysis and verification. The results show that the stress and deformation of roadway surrounding rock in inclined coal seam show obvious asymmetric distribution characteristics. The stress and deformation of roadway surrounding rock on the right side are greater than on the left side. The two sides of the roadway, the right side of the roof and the roof angle of the right side, are the key positions of roadway stress concentration and deformation. According to the variation law of stress and deformation distribution of roadway surrounding rock, roadway cyclic deformation and failure theory is put forward. The numerical simulation and physical simulation tests show that the deformation and failure law of roadway is consistent with the theoretical analysis results, and there are differences in numerical values. The cyclic deformation and failure mechanism of roadway in inclined coal seam is verified, which can provide theoretical guidance for roadway support design.

## Introduction

The reserves of inclined coal seams account for more than 35% of the total coal resources in China, and the coal quality is excellent, and the mining value is large. Under the joint influence of dip angle and various geological conditions, the surrounding rock of the inclined coal seam roadway has asymmetric deformation and failure, making the roadway's stability difficult to control and restricting the safe and efficient mining of inclined coal resources^1–6^. Therefore, the study on the deformation and failure mechanism of inclined coal seam roadway is of great significance to the design of the roadway support scheme.

The stress and deformation characteristics of roadway surrounding rock in inclined coal seam are an important basis for roadway support design. Most previous studies have simplified the roadway surrounding rock into beam, plate or arch structure for separate analysis. Rarely analyze the roof, floor and two sides of roadway surrounding rock as a whole. It is difficult to obtain the analytical solution of stress and deformation of roadway surrounding rock in inclined coal seam, so it is impossible to establish the essential relationship between roadway roof, floor and two sides of deformation. Due to the complex section of roadway, the complex function theory provides a new idea for this. The stress-deformation analysis of surrounding rock of roadway with arbitrary section can be reduced to the stress-deformation distribution problem of infinite plane with complex orifice in elasticity, which can be solved according to the complex function method.

Based on the complex function and conformal transformation theory, the complex orifice boundary can be mapped to the unit circle boundary to solve the plane problem in elasticity. The unit circle boundary corresponds to the complex orifice boundary one by one. It is easy to return the calculation results of the unit circle boundary to the original orifice boundary and obtain the solution of the complex orifice boundary. Therefore, it is widely used in the theoretical calculation of stress and deformation of cavern, tunnel, roadway, and another orifice problems^7–13^, and the analytical solution of complex orifice boundary is obtained.

At present, domestic and foreign scholars have used the complex function theory to study the stress distribution and deformation and failure law of surrounding rock of coal mine roadway. Zhao Kai, Li Ming, Shi Gaoping^14–16^ deduced the mapping function and stress calculation formula of the rectangular roadway by using conformal transformation and complex function theory and calculated the stress distribution law of surrounding rock of roadway. Xu Deqing et al.^17^ solved the mapping function of the straight-wall arched roadway in deeply inclined coal seam by conformal transformation method, deduced the formula of roadway's stress and displacement through complex function theory, and analyzed the distribution characteristics of stress and deformation of the straight-wall arched roadway. In the above studies, the complex function is used to solve the mapping function of the roadway. Still, the influence of rock dip angle and mechanical properties is not considered in the calculation process.

When solving the complex function of stress and displacement of surrounding rock of coal mine roadway, it is necessary to map the roadway section boundary to the unit circle boundary by conformal transformation. So the solution of mapping function and mapping function coefficient is very important. For the coal mine roadway in practical engineering, scholars use various methods to find the approximate mapping function. Fan Guangqin et al.^18^ proposed the absolute convergence series multiplication method to calculate the coefficients of the non-circular orifice mapping function. Lv Aizhong et al.^19,20^ used optimization technology to calculate the mapping function coefficients of orifices with arbitrary shapes. Zhu Dayong et al.^21^ proposed using the form of super long term series to approximate the mapping function analytically, but the solution is difficult, and the accuracy is low. To simplify the calculation, Chen Liang^22^ used Matlab software embedded genetic algorithm to solve the coefficients of the mapping function and obtained the analytical solution of the stress and displacement of the surrounding rock of the straight-wall arched roadway in the deeply inclined coal seam. Hu Shaoxuan^23^ also used the iterative method to solve the mapping function coefficients and considered that taking the first four order n can meet the solution accuracy of stress and deformation. Zheng Zhiqiang et al.^24^ used Schwarz–Christoffel integral to solve the mapping function coefficients, which improved the accuracy of the mapping coefficients, but the solution process was complex.

To effectively determine the mapping function's coefficient, and obtain the analytical solution of the stress and displacement of the surrounding rock of the roadway more accurately. This chapter proposes another relatively simple solution algorithm, which is based on Riemann mapping theorem and boundary correspondence principle, takes the finite term Laurent expansion, obtains the recurrence formula of Faber polynomial through Schwarz Christoffel mapping, approximates the conformal mapping function from the outer domain of any roadway section to the unit circle, solves the mapping function coefficient of inclined coal seam roadway, and introduces the inclination coefficient, The calculation formula of complex variable function of stress and displacement of surrounding rock in inclined coal seam roadway is deduced. It reveals the deformation and failure mechanism of inclined coal seam roadway. Through numerical simulation and physical similarity simulation, the deformation and failure characteristics of roadway surrounding rock are further studied, and the cyclic deformation and failure mechanism is verified, which provides theoretical guidance for the research of roadway support technology in inclined coal seam.

## Project overview

**Figure 1:**
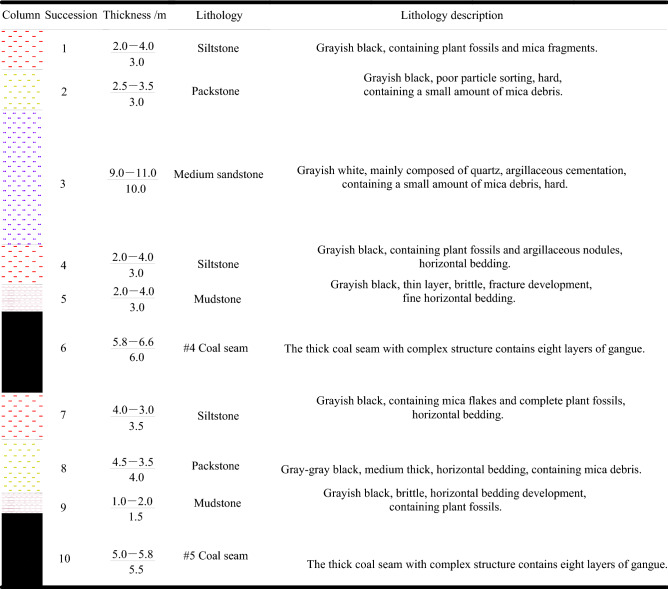
Coal stratum histogram.

The inclined coal seam in Shitanjing No. 2 mining area is thick and of good coal quality, which is of great mining value. The buried depth of the coal seam is about 405.6–480.1 m, the dip angle is between 18° and 27°, with an average of 23°, and the minable thickness is 5.8–6.6 m, with an average of 6.0 m. the coal seam contains nine layers of gangue, semi-dark type. The direct top is mudstone with a thickness of 2.0–4.0 m and an average thickness of 3.0 m. The direct bottom is siltstone with a 3.0–4.0 m and an average thickness of 3.5 m. The coal and rock stratum stress histogram is shown in Fig. 1.

**Figure 2:**
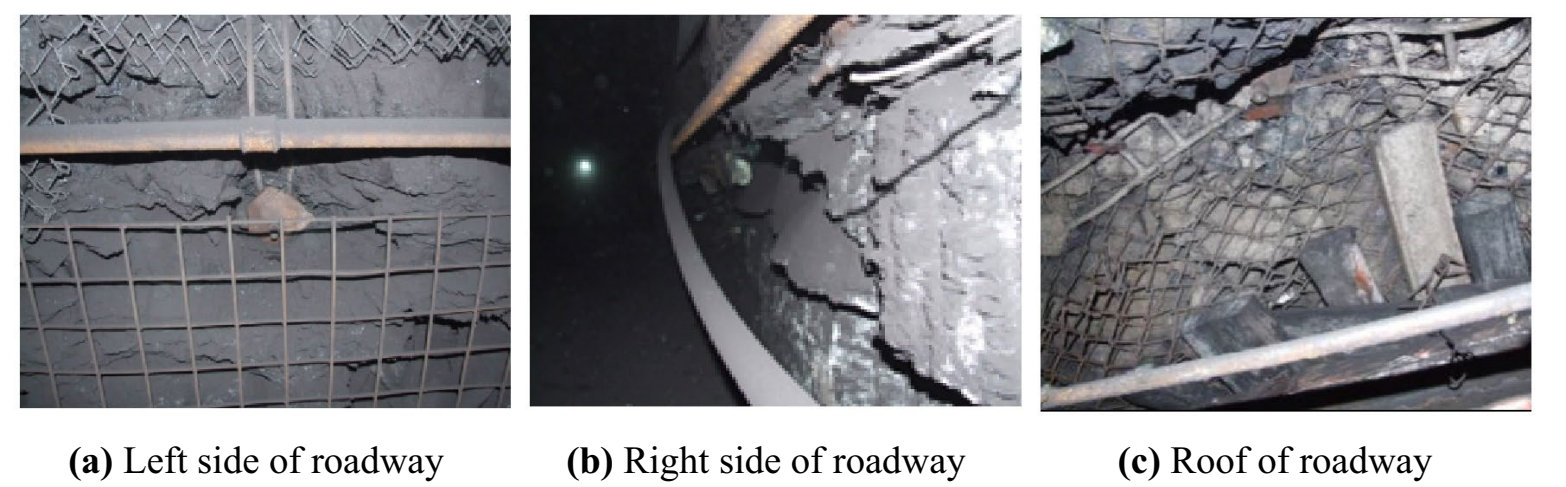
Deformation and failure of on-site roadway.

After the inclined coal seam roadway is supported by conventional anchor mesh, the surrounding rock of the roadway is still seriously deformed and damaged. It presents asymmetric characteristics, as shown in Fig. 2. Due to the influence of dip angle, section type, and in-situ stress, the two sides of the roadway have a serious slope. The deformation of the right side is greater than that of the left side. The roof sinks, the metal mesh tears, the bolt plate sinks and loosens. The bolt and anchor cable have large bending deformation, which shows that the conventional support effect is poor and brings great problems to the stability control of the surrounding rock of the roadway. To safely and economically mine inclined coal seams, it is necessary to deeply and systematically study the deformation and failure mechanism and control technology of inclined coal seam roadway.

## Complex function method to solve the stress and displacement of roadway surrounding rock

### Establishment of mechanical model

**Figure 3:**
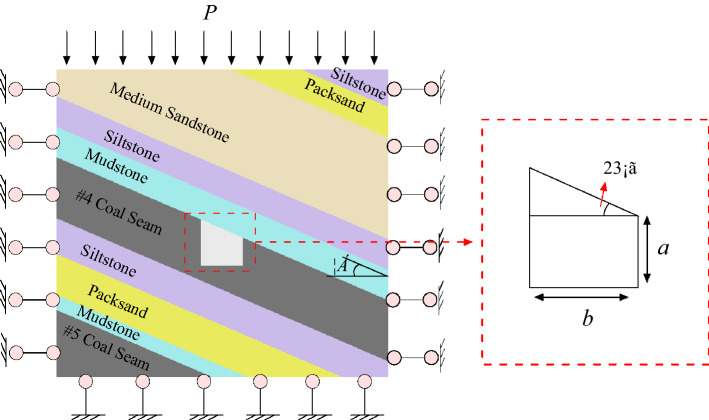
Structure diagram of inclined coal seam roadway.

Taking the inclined coal seam of the Shitanjing No. 2 mining area as the engineering background, the right-angle trapezoidal roadway is simplified into an infinite plane inner orifice problem to solve, as shown in Fig. 3. A uniform load *P* is applied to the overlying strata, the Dip angle of the coal seam is *α*, the short side height of the right-angle trapezoidal roadway is a (3.0 m), and the width is b (4.5 m). Because the roadway is located in the inclined coal seam, the stress and deformation of the surrounding rock of the roadway are affected by the asymmetric load, and the roadway is rotated by an angle of *α*, which is equivalent to the asymmetric load.

### Basic theoretical

According to the theory of complex functions and elastic mechanics, the area occupied by the roadway on the Z plane can be transformed into a unit center circle on the *ζ* plane through the mapping function z = ω(*ζ*). Assuming there are two analytical functions *φ*(*ζ*) and *ψ*(*ζ*), the stress, displacement and boundary conditions of the surrounding rock of the roadway can be expressed by them:

1 $$\left\{ \begin{gathered} \sigma_{\rho } + \sigma_{\theta } = 4{\text{Re}} \Phi (\zeta ) \hfill \\ \sigma_{\theta } - \sigma_{\rho } + 2i\tau_{\rho \theta } = \frac{{2\zeta^{2} }}{{\rho^{2} \overline{{\omega^{\prime}(\zeta )}} }}\left[ {\overline{{\omega^{\prime}(\zeta )}} \cdot \Phi^{\prime}(\zeta ) + \omega^{\prime}(\zeta )\Psi (\zeta )} \right], \hfill \\ \end{gathered} \right.$$

2 $$\frac{E}{1 + v}(u_{\rho } + iu_{\theta } ) = \frac{{\overline{\zeta } }}{\rho }\frac{{\overline{{\omega^{\prime}(\zeta )}} }}{{\left| {\omega^{\prime}(\zeta )} \right|}}\left[ {\left( {3 - 4v} \right) \, \varphi (\zeta ) - \frac{\omega (\zeta )}{{\overline{{\omega^{\prime}(\zeta )}} }}\overline{{\varphi^{\prime}(\zeta )}} - \overline{\psi (\zeta )} } \right],$$

3 $$i\int {\left( {\overline{x} + i\overline{y} } \right)} ds = \left[ {\varphi (\zeta ) + \frac{\omega (\zeta )}{{\overline{{\omega^{\prime}(\zeta )}} }}\overline{{\varphi^{\prime}(\zeta )}} + \overline{\psi (\zeta )} } \right]_{s} ,$$

where *σ*_*ρ、*_*σ*_*θ*_ and *τ*_*ρθ*_ are the radial, circumferential and tangential stresses at the points (*ρ*, *θ*) of the polar coordinate system, *u*_*ρ*_ and *u*_*θ*_ are the projections of the displacement vector on *ρ* and *θ*, *E* is the elastic modulus, *v* is poisson's ratio, and the sum of the *x* and *y* axis directions on the boundary s is $\overline{x}$ and $\overline{y}$. *ω*(*ζ*) is the mapping function, and *Φ*(*ζ*), *Ψ*(ζ), *φ*(*ζ*), *ψ*(*ζ*) are complex potential analytical functions.

4 $$\left\{ \begin{gathered} \Phi (\zeta ) = {{\varphi^{\prime}(\zeta )} \mathord{\left/ {\vphantom {{\varphi^{\prime}(\zeta )} {\omega^{\prime}(\zeta )}}} \right. \kern-\nulldelimiterspace} {\omega^{\prime}(\zeta )}} \hfill \\ \Psi (\zeta ) = {{\psi^{\prime}(\zeta )} \mathord{\left/ {\vphantom {{\psi^{\prime}(\zeta )} {\omega^{\prime}(\zeta ).}}} \right. \kern-\nulldelimiterspace} {\omega^{\prime}(\zeta ).}} \hfill \\ \end{gathered} \right.$$

The equations of *φ*(*ζ*) and *ψ*(*ζ*) on the *ζ* plane are:

5 $$\left\{ \begin{gathered} \varphi (\zeta ) = \frac{1}{{8\pi \left( {1 - v} \right)}}(\overline{X} + i\overline{Y} )\ln \zeta + \alpha \omega (\zeta ) + \varphi_{0} (\zeta ) \hfill \\ \psi (\zeta ) = - \frac{3 - 4v}{{8\pi \left( {1 - v} \right)}}(\overline{X} - i\overline{Y} )\ln \zeta + (\alpha^{\prime} + i\beta^{\prime})\omega (\zeta ) + \psi_{0} (\zeta ) \hfill \\ \alpha = \frac{1}{4}(\sigma_{1} + \sigma_{2} ),\alpha^{\prime} + i\beta^{\prime} = - \frac{1}{2}(\sigma_{1} - \sigma_{2} )e^{ - 2i\eta } \hfill \\ \varphi_{0} (\zeta ) = \sum\limits_{n = 1}^{\infty } {a_{n} \zeta^{ - n} } ,\psi_{0} (\zeta ) = \sum\limits_{n = 1}^{\infty } {b_{n} \zeta^{ - n} } , \hfill \\ \end{gathered} \right.$$

where *η* is the main stress direction, *a*_*n*_ and *b*_*n*_ are proportional constants; *φ*_*0*_(*ζ*) and *ψ*_*0*_(*ζ*) are the analytic functions of the complex; function *ζ* = *ρe*^*iθ*^ in the central unit circle; *ρ* is the distance from any point to the center of the circle, and *θ* is the angle between any point and the center of the circle in the positive *x* direction; *α*, *α*′, and *β*′ are constants related to the far field stress *σ*_1_ and *σ*_2_.

According to the stress boundary condition Eq. (3), the basic equations of *φ*_0_(*ζ*) and *ψ*_0_(*ζ*) can be obtained:

6 $$\left\{ \begin{gathered} \varphi_{0} (\zeta ) + \frac{1}{2\pi i}\int\limits_{\sigma } {\frac{\omega (\sigma )}{{\overline{{\omega^{\prime}(\sigma )}} }}\frac{{\overline{{\varphi_{0}^{\prime } (\sigma )}} }}{\sigma - \zeta }} d\sigma = - \frac{1}{2\pi i}\int\limits_{\sigma } {\frac{{h_{0} d\sigma }}{\sigma - \zeta }} \hfill \\ \psi_{0} (\zeta ) + \frac{1}{2\pi i}\int\limits_{\sigma } {\frac{{\overline{\omega (\sigma )} }}{{\omega^{\prime}(\sigma )}}\frac{{\varphi_{0}^{\prime } (\sigma )}}{\sigma - \zeta }} d\sigma = - \frac{1}{2\pi i}\int\limits_{\sigma } {\frac{{\overline{{h_{0} }} d\sigma }}{\sigma - \zeta },} \hfill \\ \end{gathered} \right.$$

7 $$\begin{aligned} h_{0} & = i\int {\left( {\overline{x} + i\overline{y} } \right)} ds - \frac{{\overline{X} + i\overline{Y} }}{2\pi }\ln \sigma - \frac{1}{{8\pi \left( {1 - v} \right)}}(\overline{X} - i\overline{Y} )\frac{\omega (\sigma )}{{\overline{{\omega^{\prime}(\sigma )}} }}\sigma \\ & \quad - 2\alpha \omega (\sigma ) - (\alpha^{\prime} - i\beta^{\prime})\overline{{\omega^{\prime}(\sigma )}} . \\ \end{aligned}$$

### Solution of mapping function coefficients

#### Theory of mapping function

Assuming that Z is a bounded connected region, its complement Z^C^ is simply connected on the extended plane. The conformal mapping function Φ can be used to map Z^C^ to the outer *ζ* plane of the unit circle, where the Laurent expansion of z is a faber polynomial, and the faber polynomial can be calculated through the recursive relationship *f*_*I*_ function to determine the coefficients of the mapping function.

1. Schwarz–Christoffel mapping function

The arbitrary conformal mapping functions from the unit circle to the inside and outside of the bounded polygon P are *f*_*I*_(z) and *f'*_*I*_(z):

8 $$f_{I} {(}z{)} = \text{A} + C_{1} \mathop \smallint \nolimits^{z} \prod\limits_{k = 1}^{n} {\left( {1 - \frac{\zeta }{{z_{k} }}} \right)^{{\alpha_{k} - 1}} } d\zeta ,$$

9 $$f^{\prime}_{I} {(}z{)} = \text{A} + C_{{1}} \mathop \smallint \nolimits^{z} \zeta^{2} \prod\limits_{k = 1}^{n} {\left( {1 - \frac{\zeta }{{z_{k} }}} \right)^{{\alpha_{k} - 1}} } d\zeta ,$$

where A and C_1_ are constants.

(2) Coefficients of faber polynomials

**Figure 4:**
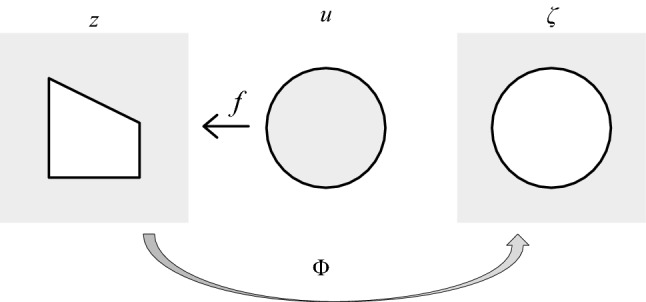
Mapping diagram.

Assuming that the complement Z^C^ of Z is simply connected on the extended plane (on the Riemannian sphere). According to Riemannian mapping theorem, there is a conformal mapping from Z^C^ to the outside of the unit circle Φ(z), so Φ(∞) = ∞, then Φ Laurent expansion:

10 $${\Phi (}z{)} = C_{{1}}^{ - 1} \left( {z + a_{{0}} + a_{{1}} z^{ - 1} + a_{{2}} z^{ - 2} + \cdots } \right),$$

where |C1|> 0, the horizontal curve of Z is *ϕ *− 1. For a circle with radius *ρ* > 1, the mapping relationsip is shown in Fig. 4.

Assuming *ζ* = Φ(z), *u* = 1/*ζ*, *f* is the Schwarz–Christoffel mapping function:

11 $$f{(}u{)} = \text{A} + C_{{1}} \mathop \smallint \nolimits^{z} \zeta^{ - 2} \prod\limits_{{k = {1}}}^{n} {\left( {1 - \frac{\zeta }{{z_{k} }}} \right)^{{1 - \alpha_{k} }} } d\zeta .$$

Then the inverse mapping of the Laurent expansion of Φ is:

12 $$z = \Phi^{ - 1} {(}\zeta {)} = C_{{1}} \zeta + C_{{0}} + C_{{2}} \zeta^{ - 1} + C_{{3}} \zeta^{ - 2} + \cdots$$

By deriving the inverse mapping of Laurent expansion and combining binomial theorem, the recursive formula of faber polynomial is obtained:

13 $$\begin{aligned} & \phi_{{0}} = {1} \\ & \phi_{{1}} = \left( {C_{1} z - C_{0} } \right) \\ & \phi_{{m + {1}}} = C_{{1}} z\phi_{m} - \left( {C_{0} \phi_{m} + \cdots + C_{m + 1} \phi_{0} } \right) - mC_{{m + {1}}} . \\ \end{aligned}$$

**Figure 5:**
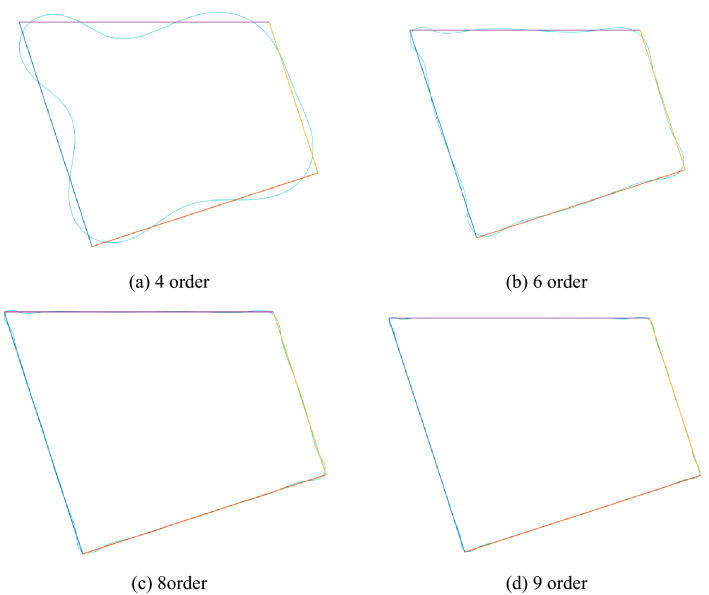
Mapping effect of roadway.

The recurrence formula of the Faber polynomial is programmed through Matlab software, and the coefficient of the mapping function is calculated up to the nth order. The mapping effect of the mapping function of different orders is shown in Fig. 5. As n increases, the average absolute error of the mapping function decreases. When n = 9, the mapping figure represented by the mapping function is very similar to the roadway section.

#### Calculation of mapping function coefficients

**Figure 6:**
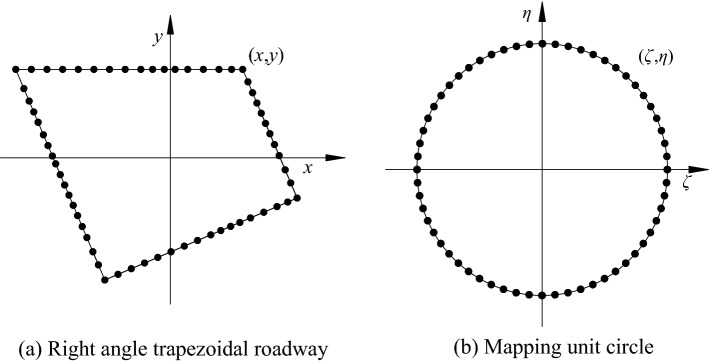
Roadway model to *ζ* mapping of plane unit circle.

The inclined coal seam roadway boundary is mapped to the unit circular boundary in the *ζ* plane through conformal transformation to solve the stress and deformation of the surrounding rock, that is, the unit circle radius *ρ* = 1, as shown in Fig. 6.

According to Laurent expansion and complex function theory, combined with the characteristics of inclined coal seam roadway (as shown in Fig. 6), the basic type of mapping function is further determined:

14 $$z = \omega {(}\zeta {)} = C_{{0}} + C_{{1}} \zeta + C_{{2}} \zeta^{{ - {1}}} + C_{{3}} \zeta^{{ - {2}}} + \cdots + C_{n} \zeta^{{ - n + {1}}} ,$$

where *C*_*j*_ (*j* = 0,1,2,3,…,n) is a complex constant, which is determined by the size and boundary shape of the roadway, and n is the number of terms of the series, so that *C*_*j*_ = *a*_*j*_ + *id*_*j*_, *z* = *x* + *iy*, *ζ* = *ρ*(cos*θ* + *i*sin*θ*) = *ρe*^*iθ*^, *ρ* = 1.

According to the Schwarz–Christoffel mapping function, combined with the recursive formula of the Faber polynomial, the calculation process of the coefficients in the mapping function is programmed through Matlab software to solve the coefficients of the roadway mapping function. In the calculation process, 1000 points are taken on the roadway section for mapping. When n is 9, the approximate roadway section obtained by the mapping is the same as the actual roadway section. The coefficients of the mapping function of the inclined coal seam roadway are shown in Table 1.

**Table 1:** Coefficients of roadway mapping function.

	*a*_*j*_	*d*_*j*_		*a*_*j*_	*d*_*j*_
*C*_0_	0.00031	0	*C*_5_	− 0.09510	− 0.11610
*C*_1_	0.00045	0.00032	*C*_6_	− 0.03114	0.02177
*C*_2_	− 0.00178	0.00137	*C*_7_	− 0.04643	− 0.05711
*C*_3_	− 0.00633	− 0.00304	*C*_8_	0.00215	− 0.02434
*C*_4_	0.01290	− 0.18520	*C*_9_	0.01932	− 0.00758

### Solution of stress and deformation of roadway surrounding rock

According to the coefficient of the mapping function, when n is 9, it is enough to meet the accuracy requirements, then the expression of the mapping function can be obtained through Eq. (14):

15 $$z = \omega {(}\zeta {)} = \left( \begin{gathered} C_{0} + C_{1} \zeta + C_{2} \zeta^{ - 1} + C_{3} \zeta^{ - 2} + C_{4} \zeta^{ - 3} + C_{5} \zeta^{ - 4} \hfill \\ + C_{6} \zeta^{ - 5} + C_{7} \zeta^{ - 6} + C_{8} \zeta^{ - 7} + C_{9} \zeta^{ - 8} \hfill \\ \end{gathered} \right).$$

According to the boundary conditions, $\overline{x}$  = $\overline{y}$ = $\overline{X}$ = $\overline{Y}$ = 0, α = *P*/2, *α'* + *iβ'* = *α'*-*iβ'* = 0, *ρ* = 1, *ζ* = *ρe*^*iθ*^ = *σ*, $\overline{\zeta }$  = *ρ*^2^/*ζ*, $\overline{\sigma }$ = 1/*σ*, then the relationship between the basic variables of each mapping function is:

16 $$\omega {(}\sigma {)} = \left( \begin{gathered} C_{{0}} + C_{{1}} \sigma + C_{{2}} \sigma^{{ - {1}}} + C_{{3}} \sigma^{{ - {2}}} + C_{{4}} \sigma^{{ - {3}}} \hfill \\ + C_{{5}} \sigma^{{ - {4}}} + C_{{6}} \sigma^{{ - {5}}} + C_{{7}} \sigma^{{ - {6}}} + C_{{8}} \sigma^{{ - {7}}} + C_{{9}} \sigma^{{ - {8}}} \hfill \\ \end{gathered} \right).$$

According to Eq. (7), we can get:

17 $$\begin{aligned} h_{{0}} & = - {2}\alpha \omega {(}\sigma {)} - {(}\alpha^{\prime} - i\beta^{\prime}{)}\overline{{\omega^{\prime}{(}\sigma {)}}} \\ & = - P\left( \begin{gathered} C_{{0}} + C_{{1}} \sigma + C_{{2}} \sigma^{{ - {1}}} + C_{{3}} \sigma^{{ - {2}}} + C_{{4}} \sigma^{{ - {3}}} \hfill \\ + C_{{5}} \sigma^{{ - {4}}} + C_{{6}} \sigma^{{ - {5}}} + C_{{7}} \sigma^{{ - {6}}} + C_{{8}} \sigma^{{ - {7}}} + C_{{9}} \sigma^{{ - {8}}} \hfill \\ \end{gathered} \right). \\ \end{aligned}$$

Substituting Eq. (17) into Eq. (6), we get

18 $$\varphi_{{0}} {(}\zeta {)} = - \frac{{1}}{{{2}\pi i}}\int\limits_{\sigma } {\frac{{h_{{0}} d\sigma }}{\sigma - \zeta }} = P\left( \begin{gathered} C_{{2}} \zeta^{{ - {1}}} + C_{{3}} \zeta^{{ - {2}}} + C_{{4}} \zeta^{{ - {3}}} \hfill \\ + C_{{5}} \zeta^{{ - {4}}} + C_{{6}} \zeta^{{ - {5}}} + C_{{7}} \zeta^{{ - {6}}} \hfill \\ + C_{{8}} \zeta^{{ - {7}}} + C_{{9}} \zeta^{{ - {8}}} \hfill \\ \end{gathered} \right),$$

19 $$\begin{aligned} \psi_{{0}} {(}\zeta {)} & = - \frac{{1}}{{{2}\pi i}}\int\limits_{\sigma } {\frac{{\overline{{h_{{0}} }} d\sigma }}{\sigma - \zeta }} - \frac{{1}}{{{2}\pi i}}\int\limits_{\sigma } {\frac{{\overline{{\omega {(}\sigma {)}}} }}{{\omega^{\prime}{(}\sigma {)}}}\frac{{\varphi_{{0}}^{\prime } {(}\sigma {)}}}{\sigma - \zeta }} d\sigma = P\left( {C_{{1}} \zeta^{{ - {1}}} } \right) \\ & \quad { + }P\frac{{\left( \begin{gathered} C_{{0}} + C_{{1}} \zeta^{{ - {1}}} + C_{{2}} \zeta + C_{{3}} \zeta^{{2}} { + }C_{{4}} \zeta^{{3}} \hfill \\ + C_{{5}} \zeta^{{4}} + C_{{6}} \zeta^{{5}} + C_{{7}} \zeta^{{6}} + C_{{8}} \zeta^{{7}} + C_{{9}} \zeta^{{8}} \hfill \\ \end{gathered} \right)}}{{\left( \begin{gathered} C_{{1}} - C_{{2}} \zeta^{{ - {2}}} - {2}C_{{3}} \zeta^{{ - {3}}} - {3}C_{{4}} \zeta^{{ - {4}}} - {4}C_{{5}} \zeta^{{ - {5}}} \hfill \\ - {5}C_{{6}} \zeta^{{ - {6}}} - {6}C_{{7}} \zeta^{{ - {7}}} - {7}C_{{8}} \zeta^{{ - {8}}} - {8}C_{{9}} \zeta^{{ - {9}}} \hfill \\ \end{gathered} \right)}} \cdot \left( \begin{gathered} - C_{\text{2}} \zeta^{{ - \text{2}}} - \text{2}C_{\text{3}} \zeta^{{ - \text{3}}} \hfill \\ - \text{3}C_{\text{4}} \zeta^{{ - \text{4}}} - \text{4}C_{\text{5}} \zeta^{{ - \text{5}}} \hfill \\ - \text{5}C_{\text{6}} \zeta^{{ - \text{6}}} - \text{6}C_{\text{7}} \zeta^{{ - \text{7}}} \hfill \\ - \text{7}C_{\text{8}} \zeta^{{ - \text{8}}} - \text{8}C_{\text{9}} \zeta^{{ - \text{9}}} \hfill \\ \end{gathered} \right). \\ \end{aligned}$$

Substituting Eqs. (18) and (19) into Eq. (5) can obtain *φ*(*ζ*) and *ψ*(*ζ*):

20 $$\varphi \text{(}\zeta \text{)} = \alpha \omega \text{(}\zeta \text{)} + \varphi_{\text{0}} \text{(}\zeta \text{) = }\frac{P}{\text{2}}\left( \begin{gathered} C_{\text{0}} + C_{\text{1}} \zeta - C_{\text{2}} \zeta^{ - 1} - C_{3} \zeta^{ - 2} \hfill \\ - C_{4} \zeta^{ - 3} - C_{5} \zeta^{ - 4} - C_{6} \zeta^{ - 5} \hfill \\ - C_{7} \zeta^{ - 6} - C_{8} \zeta^{ - 7} - C_{9} \zeta^{ - 8} \hfill \\ \end{gathered} \right),$$

21 $$\psi \text{(}\zeta \text{)} = PC_{\text{1}} \zeta^{ - 1} { + }P\frac{{\left( \begin{gathered} C_{\text{0}} + C_{\text{1}} \zeta^{{ - \text{1}}} + C_{\text{2}} \zeta + C_{\text{3}} \zeta^{\text{2}} { + }C_{\text{4}} \zeta^{\text{3}} \hfill \\ + C_{\text{5}} \zeta^{\text{4}} + C_{\text{6}} \zeta^{\text{5}} + C_{\text{7}} \zeta^{\text{6}} + C_{\text{8}} \zeta^{\text{7}} + C_{\text{9}} \zeta^{\text{8}} \hfill \\ \end{gathered} \right)}}{{\left( \begin{gathered} C_{\text{1}} - C_{\text{2}} \zeta^{{ - \text{2}}} - \text{2}C_{\text{3}} \zeta^{{ - \text{3}}} - \text{3}C_{\text{4}} \zeta^{{ - \text{4}}} - \text{4}C_{\text{5}} \zeta^{{ - \text{5}}} \hfill \\ - \text{5}C_{\text{6}} \zeta^{{ - \text{6}}} - \text{6}C_{\text{7}} \zeta^{{ - \text{7}}} - \text{7}C_{\text{8}} \zeta^{{ - \text{8}}} - \text{8}C_{\text{9}} \zeta^{{ - \text{9}}} \hfill \\ \end{gathered} \right)}} \cdot \left( \begin{gathered} - C_{\text{2}} \zeta^{{ - \text{2}}} - \text{2}C_{\text{3}} \zeta^{{ - \text{3}}} \hfill \\ - \text{3}C_{\text{4}} \zeta^{{ - \text{4}}} - \text{4}C_{\text{5}} \zeta^{{ - \text{5}}} \hfill \\ - \text{5}C_{\text{6}} \zeta^{{ - \text{6}}} - \text{6}C_{\text{7}} \zeta^{{ - \text{7}}} \hfill \\ - \text{7}C_{\text{8}} \zeta^{{ - \text{8}}} - \text{8}C_{\text{9}} \zeta^{{ - \text{9}}} \hfill \\ \end{gathered} \right).$$

Substituting Eqs. (20) and (21) into Eq. (6) can obtain *Φ*(*ζ*) and *Ψ*(*ζ*):

22 $$\Phi \text{(}\zeta \text{)} = \frac{{\varphi^{\prime}\text{(}\zeta \text{)}}}{{\omega^{\prime}\text{(}\zeta \text{)}}} = \frac{{P\left( \begin{gathered} C_{1} + C_{2} \zeta^{ - 2} + 2C_{3} \zeta^{ - 3} + 3C_{4} \zeta^{ - 4} + 4C_{5} \zeta^{*5} \hfill \\ + 5C_{6} \zeta^{ - 6} + 6C_{7} \zeta^{ - 7} + 7C_{8} \zeta^{ - 8} + 7C_{9} \zeta^{ - 9} \hfill \\ \end{gathered} \right)}}{{2\left( \begin{gathered} C_{1} - C_{2} \zeta^{ - 2} - 2C_{3} \zeta^{ - 3} - 3C_{4} \zeta^{ - 4} - 4C_{5} \zeta^{ - 5} \hfill \\ - 5C_{6} \zeta^{ - 6} - 6C_{7} \zeta^{ - 7} - 7C_{8} \zeta^{ - 8} - 8C_{9} \zeta^{ - 9} \hfill \\ \end{gathered} \right)}},$$

23 $$\begin{aligned} \Psi \text{(}\zeta \text{)} & = - PC_{\text{1}} \zeta^{{ - \text{2}}} \frac{{P\left( \begin{gathered} - C_{\text{1}} \zeta^{{ - \text{2}}} + C_{\text{2}} + \text{2}C_{\text{3}} \zeta \hfill \\ + \text{3}C_{\text{4}} \zeta^{\text{2}} + \text{4}C_{\text{5}} \zeta^{\text{3}} + \text{5}C_{\text{6}} \zeta^{\text{4}} \hfill \\ + \text{6}C_{\text{7}} \zeta^{\text{5}} + \text{7}C_{\text{8}} \zeta^{\text{6}} + \text{8}C_{\text{9}} \zeta^{\text{7}} \hfill \\ \end{gathered} \right)\left( \begin{gathered} - C_{\text{2}} \zeta^{{ - \text{2}}} - \text{2}C_{\text{3}} \zeta^{{ - \text{3}}} - \text{3}C_{\text{4}} \zeta^{{ - \text{4}}} \hfill \\ - \text{4}C_{\text{5}} \zeta^{{ - \text{5}}} - \text{5}C_{\text{6}} \zeta^{{ - \text{6}}} - \text{6}C_{\text{7}} \zeta^{{ - \text{7}}} \hfill \\ - \text{7}C_{\text{8}} \zeta^{{ - \text{8}}} - \text{8}C_{\text{9}} \zeta^{{ - \text{9}}} \hfill \\ \end{gathered} \right)}}{{\left( \begin{gathered} C_{\text{1}} - C_{\text{2}} \zeta^{{ - \text{2}}} - \text{2}C_{\text{3}} \zeta^{{ - \text{3}}} - \text{3}C_{\text{4}} \zeta^{{ - \text{4}}} - \text{42}C_{\text{5}} \zeta^{{ - \text{5}}} \hfill \\ - \text{5}C_{\text{6}} \zeta^{{ - \text{6}}} - \text{6}C_{\text{7}} \zeta^{{ - \text{7}}} - \text{7}C_{\text{8}} \zeta^{{ - \text{8}}} - \text{8}C_{\text{9}} \zeta^{{ - \text{9}}} \hfill \\ \end{gathered} \right)^{\text{2}} }} \\ & \quad + P\frac{{\left[ {\left( \begin{gathered} C_{\text{0}} + C_{\text{1}} \zeta^{{ - \text{1}}} + C_{\text{2}} \zeta \hfill \\ + C_{\text{3}} \zeta^{\text{2}} { + }C_{\text{4}} \zeta^{\text{3}} + C_{\text{5}} \zeta^{\text{4}} \hfill \\ + C_{\text{6}} \zeta^{\text{5}} + C_{\text{7}} \zeta^{\text{6}} \hfill \\ + C_{\text{8}} \zeta^{\text{7}} + C_{\text{9}} \zeta^{\text{8}} \hfill \\ \end{gathered} \right) \times \left( \begin{gathered} \text{2}C_{\text{2}} \zeta^{{ - \text{3}}} + \text{6}C_{\text{3}} \zeta^{{ - \text{4}}} \hfill \\ + \text{12}C_{\text{4}} \zeta^{{ - \text{5}}} + \text{20}C_{\text{5}} \zeta^{{ - \text{6}}} \hfill \\ + \text{30}C_{\text{6}} \zeta^{{ - \text{7}}} + \text{42}C_{\text{7}} \zeta^{{ - \text{8}}} \hfill \\ + \text{56}C_{\text{8}} \zeta^{{ - \text{9}}} + \text{72}C_{\text{9}} \zeta^{{ - \text{10}}} \hfill \\ \end{gathered} \right)} \right] \times \left( \begin{gathered} C_{\text{2}} \zeta^{{ - \text{2}}} + \text{2}C_{\text{3}} \zeta^{{ - \text{3}}} \hfill \\ + \text{3}C_{\text{4}} \zeta^{{ - \text{4}}} + \text{4}C_{\text{5}} \zeta^{{ - \text{5}}} \hfill \\ + \text{5}C_{\text{6}} \zeta^{{ - \text{6}}} + \text{6}C_{\text{7}} \zeta^{{ - \text{7}}} \hfill \\ + \text{7}C_{\text{8}} \zeta^{{ - \text{8}}} + \text{8}C_{\text{9}} \zeta^{{ - \text{9}}} \hfill \\ \end{gathered} \right)}}{{\left( {C_{\text{1}} - C_{\text{2}} \zeta^{{ - \text{2}}} - \text{2}C_{\text{3}} \zeta^{{ - \text{3}}} - \text{3}C_{\text{4}} \zeta^{{ - \text{4}}} - \text{42}C_{\text{5}} \zeta^{{ - \text{5}}} - \text{5}C_{\text{6}} \zeta^{{ - \text{6}}} - \text{6}C_{\text{7}} \zeta^{{ - \text{7}}} - \text{7}C_{\text{8}} \zeta^{{ - \text{8}}} - \text{8}C_{\text{9}} \zeta^{{ - \text{9}}} } \right)^{\text{3}} }} \\ & \quad - P\frac{{\left( \begin{gathered} C_{\text{0}} + C_{\text{1}} \zeta^{{ - \text{1}}} + C_{\text{2}} \zeta + C_{\text{3}} \zeta^{\text{2}} \hfill \\ + C_{\text{4}} \zeta^{\text{3}} + C_{\text{5}} \zeta^{\text{4}} + C_{\text{6}} \zeta^{\text{5}} \hfill \\ + C_{\text{7}} \zeta^{\text{6}} + C_{\text{8}} \zeta^{\text{7}} + C_{\text{9}} \zeta^{\text{8}} \hfill \\ \end{gathered} \right)\left( \begin{gathered} \text{2}C_{\text{2}} \zeta^{{ - \text{3}}} + \text{6}C_{\text{3}} \zeta^{{ - \text{4}}} + \text{12}C_{\text{4}} \zeta^{{ - \text{5}}} \hfill \\ + \text{20}C_{\text{5}} \zeta^{{ - \text{6}}} + \text{30}C_{\text{6}} \zeta^{{ - \text{7}}} + \text{42}C_{\text{7}} \zeta^{{ - \text{8}}} \hfill \\ + \text{56}C_{\text{8}} \zeta^{{ - \text{9}}} + \text{72}C_{\text{9}} \zeta^{{ - \text{10}}} \hfill \\ \end{gathered} \right)}}{{\left( \begin{gathered} C_{\text{1}} - C_{\text{2}} \zeta^{{ - \text{2}}} - \text{2}C_{\text{3}} \zeta^{{ - \text{3}}} - \text{3}C_{\text{4}} \zeta^{{ - \text{4}}} - \text{4}C_{\text{5}} \zeta^{{ - \text{5}}} \hfill \\ - \text{5}C_{\text{6}} \zeta^{{ - \text{6}}} - \text{6}C_{\text{7}} \zeta^{{ - \text{7}}} - \text{7}C_{\text{8}} \zeta^{{ - \text{8}}} - \text{8}C_{\text{9}} \zeta^{{ - \text{9}}} \hfill \\ \end{gathered} \right)^{\text{2}} }} - PC_{\text{1}} \zeta^{{ - \text{2}}} \\ \end{aligned}$$

Incorporating Eqs. (22) and (23) into Eq. (1) to obtain Eqs. (24) and (25), and further adding Eqs. (24) and (25) to obtain the real part of the roadway surrounding rock's hoop stress *σ*_*θ*_, radial *σ*_ρ_ and shear τ_ρ*θ*_ is Eqs. (26), and (28).

24 $$\sigma_{\rho } + \sigma_{\theta } = \text{4Re}\Phi \text{(}\zeta \text{)} = \text{2}P\text{Re}\frac{{\left( \begin{gathered} C_{1} + C_{2} \zeta^{ - 2} + 2C_{3} \zeta^{ - 3} + 3C_{4} \zeta^{ - 4} + 4C_{5} \zeta^{ - 5} \hfill \\ + 5C_{6} \zeta^{ - 6} + 6C_{7} \zeta^{ - 7} + 7C_{8} \zeta^{ - 8} + 7C_{9} \zeta^{ - 9} \hfill \\ \end{gathered} \right)}}{{\left( \begin{gathered} C_{\text{1}} - C_{\text{2}} \zeta^{{ - \text{2}}} - \text{2}C_{\text{3}} \zeta^{{ - \text{3}}} - \text{3}C_{\text{4}} \zeta^{{ - \text{4}}} - \text{4}C_{\text{5}} \zeta^{{ - \text{5}}} \hfill \\ - \text{5}C_{\text{6}} \zeta^{{ - \text{6}}} - \text{6}C_{\text{7}} \zeta^{{ - \text{7}}} - \text{7}C_{\text{8}} \zeta^{{ - \text{8}}} - \text{8}C_{\text{9}} \zeta^{{ - \text{9}}} \hfill \\ \end{gathered} \right)}},$$

25 $$\begin{aligned} \sigma_{\rho } - \sigma_{\theta } + \text{2}i\tau_{\rho \theta } & = \frac{{2\zeta^{\text{2}} }}{{\rho^{\text{2}} \overline{{\omega^{\prime}\text{(}\zeta \text{)}}} }}\left[ {\overline{{\omega^{\prime}\text{(}\zeta \text{)}}} \cdot \Phi^{\prime}\text{(}\zeta \text{)} + \omega^{\prime}\text{(}\zeta \text{)}\Psi \text{(}\zeta \text{)}} \right] \\ & = \left[ \begin{gathered} \frac{{P\left( \begin{gathered} - 2C_{2} \zeta^{ - 3} - 6C_{3} \zeta^{ - 4} - 12C_{4} \zeta^{ - 5} \hfill \\ - 20C_{5} \zeta^{ - 6} - 30C_{6} \zeta^{ - 7} - 42C_{7} \zeta^{ - 8} \hfill \\ - 56C_{8} \zeta^{ - 9} - 72C_{9} \zeta^{ - 10} \hfill \\ \end{gathered} \right) \cdot \left( \begin{gathered} - 2C_{2} \zeta - 4C_{3} \zeta^{ - 1} - 6C_{4} \zeta^{ - 2} \hfill \\ - 8C_{5} \zeta^{ - 3} - 10C_{6} \zeta^{ - 4} - 12C_{7} \zeta^{ - 5} \hfill \\ - 14C_{8} \zeta^{ - 6} - 16C_{9} \zeta^{ - 7} \hfill \\ \end{gathered} \right)}}{{\left( \begin{gathered} C_{\text{1}} - C_{\text{2}} \zeta^{{ - \text{2}}} - \text{2}C_{\text{3}} \zeta^{{ - \text{3}}} - \text{3}C_{\text{4}} \zeta^{{ - \text{4}}} - \text{4}C_{\text{5}} \zeta^{{ - \text{5}}} \hfill \\ - \text{5}C_{\text{6}} \zeta^{{ - \text{6}}} - \text{6}C_{\text{7}} \zeta^{{ - \text{7}}} - \text{7}C_{\text{8}} \zeta^{{ - \text{8}}} - \text{8}C_{\text{9}} \zeta^{{ - \text{9}}} \hfill \\ \end{gathered} \right)^{\text{2}} }} \hfill \\ + P\frac{{C_{\text{1}} \left( \begin{gathered} \text{2}C_{\text{1}} \zeta - \text{2}C_{\text{2}} \zeta^{{ - \text{1}}} - \text{4}C_{\text{3}} \zeta^{{ - \text{2}}} \hfill \\ - \text{6}C_{\text{4}} \zeta^{{ - \text{3}}} - \text{8}C_{\text{5}} \zeta^{{ - \text{4}}} \hfill \\ - \text{10}C_{\text{6}} \zeta^{{ - \text{5}}} - \text{12}C_{\text{7}} \zeta^{{ - \text{6}}} \hfill \\ - \text{14}C_{\text{8}} \zeta^{{ - \text{7}}} - \text{16}C_{\text{9}} \zeta^{{ - \text{8}}} \hfill \\ \end{gathered} \right){ + }\text{2}\left( \begin{gathered} C_{\text{0}} + C_{\text{1}} \zeta^{{ - \text{1}}} + C_{\text{2}} \zeta \hfill \\ + C_{\text{3}} \zeta^{\text{2}} { + }C_{\text{4}} \zeta^{\text{3}} + C_{\text{5}} \zeta^{\text{4}} \hfill \\ + C_{\text{6}} \zeta^{\text{5}} + C_{\text{7}} \zeta^{\text{6}} \hfill \\ + C_{\text{8}} \zeta^{\text{7}} + C_{\text{9}} \zeta^{\text{8}} \hfill \\ \end{gathered} \right) \cdot \left( \begin{gathered} - C_{\text{2}} - \text{2}C_{\text{3}} \zeta^{{ - \text{1}}} \hfill \\ - \text{3}C_{\text{4}} \zeta^{{ - \text{2}}} - \text{4}C_{\text{5}} \zeta^{{ - \text{3}}} \hfill \\ - \text{5}C_{\text{6}} \zeta^{{ - \text{4}}} - \text{6}C_{\text{7}} \zeta^{{ - \text{5}}} \hfill \\ - \text{7}C_{\text{8}} \zeta^{{ - \text{6}}} - \text{8}C_{\text{9}} \zeta^{{ - \text{7}}} \hfill \\ \end{gathered} \right)}}{{\left( \begin{gathered} C_{\text{1}} - C_{\text{2}} \zeta^{\text{2}} - \text{2}C_{\text{3}} \zeta^{\text{3}} \hfill \\ - \text{3}C_{\text{4}} \zeta^{\text{4}} - \text{4}C_{\text{5}} \zeta^{\text{5}} - \text{5}C_{\text{6}} \zeta^{\text{6}} \hfill \\ - \text{6}C_{\text{7}} \zeta^{\text{7}} - \text{7}C_{\text{8}} \zeta^{\text{8}} - \text{8}C_{\text{9}} \zeta^{\text{9}} \hfill \\ \end{gathered} \right)}} \hfill \\ \end{gathered} \right] \\ \end{aligned}$$

26 $$\sigma_{\theta } = P\text{Re}\left[ \begin{gathered} \frac{{\left( \begin{gathered} C_{\text{1}} + C_{\text{2}} \zeta^{{ - \text{2}}} + \text{2}C_{\text{3}} \zeta^{{ - \text{3}}} \hfill \\ + \text{3}C_{\text{4}} \zeta^{{ - \text{4}}} + \text{4}C_{\text{5}} \zeta^{{ - \text{5}}} \hfill \\ + \text{5}C_{\text{6}} \zeta^{{ - \text{6}}} + \text{6}C_{\text{7}} \zeta^{{ - \text{7}}} \hfill \\ + \text{7}C_{\text{8}} \zeta^{{ - \text{8}}} + \text{7}C_{\text{9}} \zeta^{{ - \text{9}}} \hfill \\ \end{gathered} \right)}}{{\left( \begin{gathered} C_{\text{1}} - C_{\text{2}} \zeta^{{ - \text{2}}} - \text{2}C_{\text{3}} \zeta^{{ - \text{3}}} \hfill \\ - \text{3}C_{\text{4}} \zeta^{{ - \text{4}}} - \text{4}C_{\text{5}} \zeta^{{ - \text{5}}} \hfill \\ - \text{5}C_{\text{6}} \zeta^{{ - \text{6}}} - \text{6}C_{\text{7}} \zeta^{{ - \text{7}}} \hfill \\ - \text{7}C_{\text{8}} \zeta^{{ - \text{8}}} - \text{8}C_{\text{9}} \zeta^{{ - \text{9}}} \hfill \\ \end{gathered} \right)}} - \frac{{\left( \begin{gathered} - \text{2}C_{\text{2}} \zeta^{{ - \text{3}}} - \text{6}C_{\text{3}} \zeta^{{ - \text{4}}} \hfill \\ - \text{12}C_{\text{4}} \zeta^{{ - \text{5}}} - \text{20}C_{\text{5}} \zeta^{{ - \text{6}}} \hfill \\ - \text{30}C_{\text{6}} \zeta^{{ - \text{7}}} - \text{42}C_{\text{7}} \zeta^{{ - \text{8}}} \hfill \\ - \text{56}C_{\text{8}} \zeta^{{ - \text{9}}} - \text{72}C_{\text{9}} \zeta^{{ - \text{10}}} \hfill \\ \end{gathered} \right) \cdot \left( \begin{gathered} - C_{\text{2}} \zeta - \text{2}C_{\text{3}} \zeta^{{ - \text{1}}} \hfill \\ - \text{3}C_{\text{4}} \zeta^{{ - \text{2}}} - \text{4}C_{\text{5}} \zeta^{{ - \text{3}}} \hfill \\ - \text{5}C_{\text{6}} \zeta^{{ - \text{4}}} - \text{6}C_{\text{7}} \zeta^{{ - \text{5}}} \hfill \\ - \text{7}C_{\text{8}} \zeta^{{ - \text{6}}} - \text{8}C_{\text{9}} \zeta^{{ - \text{7}}} \hfill \\ \end{gathered} \right)}}{{\left( \begin{gathered} C_{\text{1}} - C_{\text{2}} \zeta^{{ - \text{2}}} - \text{2}C_{\text{3}} \zeta^{{ - \text{3}}} \hfill \\ - \text{3}C_{\text{4}} \zeta^{{ - \text{4}}} - \text{4}C_{\text{5}} \zeta^{{ - \text{5}}} \hfill \\ - \text{5}C_{\text{6}} \zeta^{{ - \text{6}}} - \text{6}C_{\text{7}} \zeta^{{ - \text{7}}} \hfill \\ - \text{7}C_{\text{8}} \zeta^{{ - \text{8}}} - \text{8}C_{\text{9}} \zeta^{{ - \text{9}}} \hfill \\ \end{gathered} \right)^{\text{2}} }} \hfill \\ - \frac{{C_{\text{1}} \left( \begin{gathered} C_{\text{1}} \zeta - C_{\text{2}} \zeta^{{ - \text{1}}} \hfill \\ - \text{2}C_{\text{3}} \zeta^{{ - \text{2}}} - \text{3}C_{\text{4}} \zeta^{{ - \text{3}}} \hfill \\ - \text{4}C_{\text{5}} \zeta^{{ - \text{4}}} - \text{5}C_{\text{6}} \zeta^{{ - \text{5}}} \hfill \\ - \text{6}C_{\text{7}} \zeta^{{ - \text{6}}} - \text{7}C_{\text{8}} \zeta^{{ - \text{7}}} \hfill \\ - \text{8}C_{\text{9}} \zeta^{{ - \text{8}}} \hfill \\ \end{gathered} \right){ + }\left( \begin{gathered} C_{\text{0}} + C_{\text{1}} \zeta^{{ - \text{1}}} \hfill \\ + C_{\text{2}} \zeta + C_{\text{3}} \zeta^{\text{2}} \hfill \\ { + }C_{\text{4}} \zeta^{\text{3}} + C_{\text{5}} \zeta^{\text{4}} \hfill \\ + C_{\text{6}} \zeta^{\text{5}} + C_{\text{7}} \zeta^{\text{6}} \hfill \\ + C_{\text{8}} \zeta^{\text{7}} + C_{\text{9}} \zeta^{\text{8}} \hfill \\ \end{gathered} \right) \cdot \left( \begin{gathered} - C_{\text{2}} - \text{2}C_{\text{3}} \zeta^{{ - \text{1}}} \hfill \\ - \text{3}C_{\text{4}} \zeta^{{ - \text{2}}} - \text{4}C_{\text{5}} \zeta^{{ - \text{3}}} \hfill \\ - \text{5}C_{\text{6}} \zeta^{{ - \text{4}}} - \text{6}C_{\text{7}} \zeta^{{ - \text{5}}} \hfill \\ - \text{7}C_{\text{8}} \zeta^{{ - \text{6}}} - \text{8}C_{\text{9}} \zeta^{{ - \text{7}}} \hfill \\ \end{gathered} \right)}}{{\left( \begin{gathered} C_{\text{1}} - C_{\text{2}} \zeta^{\text{2}} - \text{2}C_{\text{3}} \zeta^{\text{3}} \hfill \\ - \text{3}C_{\text{4}} \zeta^{\text{4}} - \text{4}C_{\text{5}} \zeta^{\text{5}} - \text{5}C_{\text{6}} \zeta^{\text{6}} \hfill \\ - \text{6}C_{\text{7}} \zeta^{\text{7}} - \text{7}C_{\text{8}} \zeta^{\text{8}} - \text{8}C_{\text{9}} \zeta^{\text{9}} \hfill \\ \end{gathered} \right)}} \hfill \\ \end{gathered} \right],$$

27 $$\sigma_{\rho } = P\text{Re}\left[ \begin{gathered} \frac{{\left( \begin{gathered} C_{\text{1}} + C_{\text{2}} \zeta^{{ - \text{2}}} + \text{2}C_{\text{3}} \zeta^{{ - \text{3}}} \hfill \\ + \text{3}C_{\text{4}} \zeta^{{ - \text{4}}} + \text{4}C_{\text{5}} \zeta^{{ - \text{5}}} \hfill \\ + \text{5}C_{\text{6}} \zeta^{{ - \text{6}}} + \text{6}C_{\text{7}} \zeta^{{ - \text{7}}} \hfill \\ + \text{7}C_{\text{8}} \zeta^{{ - \text{8}}} + \text{7}C_{\text{9}} \zeta^{{ - \text{9}}} \hfill \\ \end{gathered} \right)}}{{\left( \begin{gathered} C_{\text{1}} - C_{\text{2}} \zeta^{{ - \text{2}}} - \text{2}C_{\text{3}} \zeta^{{ - \text{3}}} \hfill \\ - \text{3}C_{\text{4}} \zeta^{{ - \text{4}}} - \text{4}C_{\text{5}} \zeta^{{ - \text{5}}} \hfill \\ - \text{5}C_{\text{6}} \zeta^{{ - \text{6}}} - \text{6}C_{\text{7}} \zeta^{{ - \text{7}}} \hfill \\ - \text{7}C_{\text{8}} \zeta^{{ - \text{8}}} - \text{8}C_{\text{9}} \zeta^{{ - \text{9}}} \hfill \\ \end{gathered} \right)}} + \frac{{\left( \begin{gathered} - \text{2}C_{\text{2}} \zeta^{{ - \text{3}}} - \text{6}C_{\text{3}} \zeta^{{ - \text{4}}} \hfill \\ - \text{12}C_{\text{4}} \zeta^{{ - \text{5}}} - \text{20}C_{\text{5}} \zeta^{{ - \text{6}}} \hfill \\ - \text{30}C_{\text{6}} \zeta^{{ - \text{7}}} - \text{42}C_{\text{7}} \zeta^{{ - \text{8}}} \hfill \\ - \text{56}C_{\text{8}} \zeta^{{ - \text{9}}} - \text{72}C_{\text{9}} \zeta^{{ - \text{10}}} \hfill \\ \end{gathered} \right) \cdot \left( \begin{gathered} - C_{\text{2}} \zeta - \text{2}C_{\text{3}} \zeta^{{ - \text{1}}} \hfill \\ - \text{3}C_{\text{4}} \zeta^{{ - \text{2}}} - \text{4}C_{\text{5}} \zeta^{{ - \text{3}}} \hfill \\ - \text{5}C_{\text{6}} \zeta^{{ - \text{4}}} - \text{6}C_{\text{7}} \zeta^{{ - \text{5}}} \hfill \\ - \text{7}C_{\text{8}} \zeta^{{ - \text{6}}} - \text{8}C_{\text{9}} \zeta^{{ - \text{7}}} \hfill \\ \end{gathered} \right)}}{{\left( \begin{gathered} C_{\text{1}} - C_{\text{2}} \zeta^{{ - \text{2}}} - \text{2}C_{\text{3}} \zeta^{{ - \text{3}}} \hfill \\ - \text{3}C_{\text{4}} \zeta^{{ - \text{4}}} - \text{4}C_{\text{5}} \zeta^{{ - \text{5}}} \hfill \\ - \text{5}C_{\text{6}} \zeta^{{ - \text{6}}} - \text{6}C_{\text{7}} \zeta^{{ - \text{7}}} \hfill \\ - \text{7}C_{\text{8}} \zeta^{{ - \text{8}}} - \text{8}C_{\text{9}} \zeta^{{ - \text{9}}} \hfill \\ \end{gathered} \right)^{\text{2}} }} \hfill \\ + \frac{{C_{\text{1}} \left( \begin{gathered} C_{\text{1}} \zeta - C_{\text{2}} \zeta^{{ - \text{1}}} - \text{2}C_{\text{3}} \zeta^{{ - \text{2}}} \hfill \\ - \text{3}C_{\text{4}} \zeta^{{ - \text{3}}} - \text{4}C_{\text{5}} \zeta^{{ - \text{4}}} \hfill \\ - \text{5}C_{\text{6}} \zeta^{{ - \text{5}}} - \text{6}C_{\text{7}} \zeta^{{ - \text{6}}} \hfill \\ - \text{7}C_{\text{8}} \zeta^{{ - \text{7}}} - \text{8}C_{\text{9}} \zeta^{{ - \text{8}}} \hfill \\ \end{gathered} \right){ + }\left( \begin{gathered} C_{\text{0}} + C_{\text{1}} \zeta^{{ - \text{1}}} \hfill \\ + C_{\text{2}} \zeta + C_{\text{3}} \zeta^{\text{2}} \hfill \\ { + }C_{\text{4}} \zeta^{\text{3}} + C_{\text{5}} \zeta^{\text{4}} \hfill \\ + C_{\text{6}} \zeta^{\text{5}} + C_{\text{7}} \zeta^{\text{6}} \hfill \\ + C_{\text{8}} \zeta^{\text{7}} + C_{\text{9}} \zeta^{\text{8}} \hfill \\ \end{gathered} \right) \cdot \left( \begin{gathered} - C_{\text{2}} - \text{2}C_{\text{3}} \zeta^{{ - \text{1}}} \hfill \\ - \text{3}C_{\text{4}} \zeta^{{ - \text{2}}} - \text{4}C_{\text{5}} \zeta^{{ - \text{3}}} \hfill \\ - \text{5}C_{\text{6}} \zeta^{{ - \text{4}}} - \text{6}C_{\text{7}} \zeta^{{ - \text{5}}} \hfill \\ - \text{7}C_{\text{8}} \zeta^{{ - \text{6}}} - \text{8}C_{\text{9}} \zeta^{{ - \text{7}}} \hfill \\ \end{gathered} \right)}}{{\left( \begin{gathered} C_{\text{1}} - C_{\text{2}} \zeta^{\text{2}} - \text{2}C_{\text{3}} \zeta^{\text{3}} \hfill \\ - \text{3}C_{\text{4}} \zeta^{\text{4}} - \text{4}C_{\text{5}} \zeta^{\text{5}} - \text{5}C_{\text{6}} \zeta^{\text{6}} \hfill \\ - \text{6}C_{\text{7}} \zeta^{\text{7}} - \text{7}C_{\text{8}} \zeta^{\text{8}} - \text{8}C_{\text{9}} \zeta^{\text{9}} \hfill \\ \end{gathered} \right)}} \hfill \\ \end{gathered} \right],$$

28 $$\tau_{\rho \theta } = P\text{Im}\left[ \begin{gathered} \frac{{\left( \begin{gathered} - \text{2}C_{\text{2}} \zeta^{{ - \text{3}}} - \text{6}C_{\text{3}} \zeta^{{ - \text{4}}} - \text{12}C_{\text{4}} \zeta^{{ - \text{5}}} \hfill \\ - \text{20}C_{\text{5}} \zeta^{{ - \text{6}}} - \text{30}C_{\text{6}} \zeta^{{ - \text{7}}} - \text{42}C_{\text{7}} \zeta^{{ - \text{8}}} \hfill \\ - \text{56}C_{\text{8}} \zeta^{{ - \text{9}}} - \text{72}C_{\text{9}} \zeta^{{ - \text{10}}} \hfill \\ \end{gathered} \right) \cdot \left( \begin{gathered} - \text{2}C_{\text{2}} \zeta - \text{4}C_{\text{3}} \zeta^{{ - \text{1}}} - \text{6}C_{\text{4}} \zeta^{{ - \text{2}}} \hfill \\ - \text{8}C_{\text{5}} \zeta^{{ - \text{3}}} - \text{10}C_{\text{6}} \zeta^{{ - \text{4}}} - \text{12}C_{\text{7}} \zeta^{{ - \text{5}}} \hfill \\ - \text{14}C_{\text{8}} \zeta^{{ - \text{6}}} - \text{16}C_{\text{9}} \zeta^{{ - \text{7}}} \hfill \\ \end{gathered} \right)}}{{\text{2}\left( \begin{gathered} C_{\text{1}} - C_{\text{2}} \zeta^{{ - \text{2}}} - \text{2}C_{\text{3}} \zeta^{{ - \text{3}}} - \text{3}C_{\text{4}} \zeta^{{ - \text{4}}} - \text{4}C_{\text{5}} \zeta^{{ - \text{5}}} \hfill \\ - \text{5}C_{\text{6}} \zeta^{{ - \text{6}}} - \text{6}C_{\text{7}} \zeta^{{ - \text{7}}} - \text{7}C_{\text{8}} \zeta^{{ - \text{8}}} - \text{8}C_{\text{9}} \zeta^{{ - \text{9}}} \hfill \\ \end{gathered} \right)^{\text{2}} }} + \hfill \\ \frac{{C_{\text{1}} \left( \begin{gathered} \text{2}C_{\text{1}} \zeta - \text{2}C_{\text{2}} \zeta^{{ - \text{1}}} \hfill \\ - \text{4}C_{\text{3}} \zeta^{{ - \text{2}}} - \text{6}C_{\text{4}} \zeta^{{ - \text{3}}} \hfill \\ - \text{8}C_{\text{5}} \zeta^{{ - \text{4}}} - \text{10}C_{\text{6}} \zeta^{{ - \text{5}}} \hfill \\ - \text{12}C_{\text{7}} \zeta^{{ - \text{6}}} - \text{14}C_{\text{8}} \zeta^{{ - \text{7}}} \hfill \\ - \text{16}C_{\text{9}} \zeta^{{ - \text{8}}} \hfill \\ \end{gathered} \right){ + }\text{2}\left( \begin{gathered} C_{\text{0}} + C_{\text{1}} \zeta^{{ - \text{1}}} \hfill \\ + C_{\text{2}} \zeta + C_{\text{3}} \zeta^{\text{2}} \hfill \\ { + }C_{\text{4}} \zeta^{\text{3}} + C_{\text{5}} \zeta^{\text{4}} \hfill \\ + C_{\text{6}} \zeta^{\text{5}} + C_{\text{7}} \zeta^{\text{6}} \hfill \\ + C_{\text{8}} \zeta^{\text{7}} + C_{\text{9}} \zeta^{\text{8}} \hfill \\ \end{gathered} \right) \cdot \left( \begin{gathered} - C_{\text{2}} - \text{2}C_{\text{3}} \zeta^{{ - \text{1}}} \hfill \\ - \text{3}C_{\text{4}} \zeta^{{ - \text{2}}} - \text{4}C_{\text{5}} \zeta^{{ - \text{3}}} \hfill \\ - \text{5}C_{\text{6}} \zeta^{{ - \text{4}}} - \text{6}C_{\text{7}} \zeta^{{ - \text{5}}} \hfill \\ - \text{7}C_{\text{8}} \zeta^{{ - \text{6}}} - \text{8}C_{\text{9}} \zeta^{{ - \text{7}}} \hfill \\ \end{gathered} \right)}}{{\text{2}\left( \begin{gathered} C_{\text{1}} - C_{\text{2}} \zeta^{\text{2}} - \text{2}C_{\text{3}} \zeta^{\text{3}} - \text{3}C_{\text{4}} \zeta^{\text{4}} - \text{4}C_{\text{5}} \zeta^{\text{5}} \hfill \\ - \text{5}C_{\text{6}} \zeta^{\text{6}} - \text{6}C_{\text{7}} \zeta^{\text{7}} - \text{7}C_{\text{8}} \zeta^{\text{8}} - \text{8}C_{\text{9}} \zeta^{\text{9}} \hfill \\ \end{gathered} \right)}} \hfill \\ \end{gathered} \right],$$

Substituting Eqs. (22) and (23) into Eq. (2) and taking the real part, the radial displacement *u*_*ρ*_ and radial displacement *u*_ρ_ of the surrounding rock of the inclined coal seam roadway can be obtained as:

29 $$\begin{aligned} u_{\rho } & = \text{Re}\left\{ {\frac{{\overline{\zeta } }}{\rho }\frac{{\overline{{\omega^{\prime}\text{(}\zeta \text{)}}} }}{{\left| {\omega^{\prime}\text{(}\zeta \text{)}} \right|}}\left[ {\frac{{\text{3} - v}}{{\text{1} + v}}\varphi \text{(}\zeta \text{)} - \overline{{\psi \text{(}\zeta \text{)}}} - \frac{{\omega \text{(}\zeta \text{)}}}{{\overline{{\omega^{\prime}\text{(}\zeta \text{)}}} }}\overline{{\varphi^{\prime}\text{(}\zeta \text{)}}} } \right]} \right\} = \frac{{P\left( {\text{1} + v} \right)}}{E} \\ & \quad \times \text{Re}\left\{ {\frac{{\text{3} - v}}{{\text{2}\left( {\text{1} + v} \right)}}\frac{{\left( \begin{gathered} C_{\text{0}} + C_{\text{1}} \zeta - C_{\text{2}} \zeta^{{ - \text{1}}} - C_{\text{3}} \zeta^{{ - \text{2}}} \hfill \\ - C_{\text{4}} \zeta^{{ - \text{3}}} - C_{\text{5}} \zeta^{{ - \text{4}}} - C_{\text{6}} \zeta^{{ - \text{5}}} \hfill \\ - C_{\text{7}} \zeta^{{ - \text{6}}} - C_{\text{8}} \zeta^{{ - \text{7}}} - C_{\text{9}} \zeta^{{ - \text{8}}} \hfill \\ \end{gathered} \right)}}{{\left( \begin{gathered} C_{\text{1}} \zeta - C_{\text{2}} \zeta^{{ - \text{1}}} - \text{2}C_{\text{3}} \zeta^{{ - \text{2}}} \hfill \\ - \text{3}C_{\text{4}} \zeta^{{ - \text{3}}} - \text{42}C_{\text{5}} \zeta^{{ - \text{4}}} - \text{5}C_{\text{6}} \zeta^{{ - \text{5}}} \hfill \\ - \text{6}C_{\text{7}} \zeta^{{ - \text{6}}} - \text{7}C_{\text{8}} \zeta^{{ - \text{7}}} - \text{8}C_{\text{9}} \zeta^{{ - \text{8}}} \hfill \\ \end{gathered} \right)}}{ + }\left[ \begin{gathered} \left( \begin{gathered} C_{\text{0}} + C_{\text{1}} \zeta + C_{\text{2}} \zeta^{{ - \text{1}}} + C_{\text{3}} \zeta^{{ - \text{2}}} \hfill \\ { + }C_{\text{4}} \zeta^{{ - \text{3}}} + C_{\text{5}} \zeta^{{ - \text{4}}} + C_{\text{6}} \zeta^{{ - \text{5}}} \hfill \\ + C_{\text{7}} \zeta^{{ - \text{6}}} + C_{\text{8}} \zeta^{{ - \text{7}}} + C_{\text{9}} \zeta^{{ - \text{8}}} \hfill \\ \end{gathered} \right) \hfill \\ \times \left( \begin{gathered} - C_{\text{2}} \zeta^{\text{1}} - \text{2}C_{\text{3}} \zeta^{\text{2}} - \text{3}C_{\text{4}} \zeta^{\text{3}} \hfill \\ - \text{4}C_{\text{5}} \zeta^{\text{4}} - \text{5}C_{\text{6}} \zeta^{\text{5}} - \text{6}C_{\text{7}} \zeta^{\text{6}} \hfill \\ - \text{7}C_{\text{8}} \zeta^{\text{7}} - \text{8}C_{\text{9}} \zeta^{\text{8}} \hfill \\ \end{gathered} \right) \hfill \\ \end{gathered} \right]} \right\} \\ & \quad - \frac{{P\left( {\text{1} + v} \right)}}{E}\text{Re}\left[ {C_{\text{1}} + \frac{{\left( \begin{gathered} C_{\text{0}} + C_{\text{1}} \zeta + C_{\text{2}} \zeta^{{ - \text{1}}} + C_{\text{3}} \zeta^{{ - \text{2}}} \hfill \\ + C_{\text{4}} \zeta^{{ - \text{3}}} + C_{\text{5}} \zeta^{{ - \text{4}}} + C_{\text{6}} \zeta^{{ - \text{5}}} \hfill \\ + C_{\text{7}} \zeta^{{ - \text{6}}} + C_{\text{8}} \zeta^{{ - \text{7}}} + C_{\text{9}} \zeta^{{ - \text{8}}} \hfill \\ \end{gathered} \right)}}{{\text{2}\left( \begin{gathered} C_{\text{1}} \zeta - C_{\text{2}} \zeta^{{ - \text{1}}} - \text{2}C_{\text{3}} \zeta^{{ - \text{2}}} \hfill \\ - \text{3}C_{\text{4}} \zeta^{{ - \text{3}}} - \text{42}C_{\text{5}} \zeta^{{ - \text{4}}} - \text{5}C_{\text{6}} \zeta^{{ - \text{5}}} \hfill \\ - \text{6}C_{\text{7}} \zeta^{{ - \text{6}}} - \text{7}C_{\text{8}} \zeta^{{ - \text{7}}} - \text{8}C_{\text{9}} \zeta^{{ - \text{8}}} \hfill \\ \end{gathered} \right)}} \times \left( \begin{gathered} C_{\text{1}} + C_{\text{2}} \zeta^{\text{2}} + \text{2}C_{\text{3}} \zeta^{\text{3}} \hfill \\ + \text{3}C_{\text{4}} \zeta^{\text{4}} + \text{4}C_{\text{5}} \zeta^{\text{5}} \hfill \\ + \text{5}C_{\text{6}} \zeta^{\text{6}} + \text{6}C_{\text{7}} \zeta^{\text{7}} \hfill \\ + \text{7}C_{\text{8}} \zeta^{\text{8}} + \text{8}C_{\text{9}} \zeta^{\text{9}} \hfill \\ \end{gathered} \right)} \right]. \\ \\ \end{aligned}$$

30 $$\begin{aligned} u_{\theta } & = \text{Im}\left\{ {\frac{{\overline{\zeta } }}{\rho }\frac{{\overline{{\omega^{\prime}\text{(}\zeta \text{)}}} }}{{\left| {\omega^{\prime}\text{(}\zeta \text{)}} \right|}}\left[ {\frac{{\text{3} - v}}{{\text{1} + v}}\varphi \text{(}\zeta \text{)} - \overline{{\psi \text{(}\zeta \text{)}}} - \frac{{\omega \text{(}\zeta \text{)}}}{{\overline{{\omega^{\prime}\text{(}\zeta \text{)}}} }}\overline{{\varphi^{\prime}\text{(}\zeta \text{)}}} } \right]} \right\} = \frac{{P\left( {\text{1} + v} \right)}}{E} \\ & \quad \times \text{Im}\left\{ {\frac{{\text{3} - v}}{{\text{2}\left( {\text{1} + v} \right)}}\frac{{\left( \begin{gathered} C_{\text{0}} + C_{\text{1}} \zeta - C_{\text{2}} \zeta^{{\text{ - 1}}} - C_{\text{3}} \zeta^{{\text{ - 2}}} \hfill \\ - C_{\text{4}} \zeta^{{\text{ - 3}}} - C_{\text{5}} \zeta^{{\text{ - 4}}} - C_{\text{6}} \zeta^{{\text{ - 5}}} \hfill \\ - C_{\text{7}} \zeta^{{\text{ - 6}}} - C_{\text{8}} \zeta^{{\text{ - 7}}} - C_{\text{9}} \zeta^{{\text{ - 8}}} \hfill \\ \end{gathered} \right)}}{{\left( \begin{gathered} C_{\text{1}} \zeta - C_{\text{2}} \zeta^{{ - \text{1}}} - \text{2}C_{\text{3}} \zeta^{{ - \text{2}}} \hfill \\ - \text{3}C_{\text{4}} \zeta^{{ - \text{3}}} - \text{42}C_{\text{5}} \zeta^{{ - \text{4}}} - \text{5}C_{\text{6}} \zeta^{{ - \text{5}}} \hfill \\ - \text{6}C_{\text{7}} \zeta^{{ - \text{6}}} - \text{7}C_{\text{8}} \zeta^{{ - \text{7}}} - \text{8}C_{\text{9}} \zeta^{{ - \text{8}}} \hfill \\ \end{gathered} \right)}}{ + }\left[ \begin{gathered} \left( \begin{gathered} C_{\text{0}} + C_{\text{1}} \zeta + C_{\text{2}} \zeta^{{ - \text{1}}} + C_{\text{3}} \zeta^{{ - \text{2}}} \hfill \\ { + }C_{\text{4}} \zeta^{{ - \text{3}}} + C_{\text{5}} \zeta^{{ - \text{4}}} + C_{\text{6}} \zeta^{{ - \text{5}}} \hfill \\ + C_{\text{7}} \zeta^{{ - \text{6}}} + C_{\text{8}} \zeta^{{ - \text{7}}} + C_{\text{9}} \zeta^{{ - \text{8}}} \hfill \\ \end{gathered} \right) \hfill \\ \times \left( \begin{gathered} - C_{\text{2}} \zeta^{\text{1}} - \text{2}C_{\text{3}} \zeta^{\text{2}} - \text{3}C_{\text{4}} \zeta^{\text{3}} \hfill \\ - \text{4}C_{\text{5}} \zeta^{\text{4}} - \text{5}C_{\text{6}} \zeta^{\text{5}} - \text{6}C_{\text{7}} \zeta^{\text{6}} \hfill \\ - \text{7}C_{\text{8}} \zeta^{\text{7}} - \text{8}C_{\text{9}} \zeta^{\text{8}} \hfill \\ \end{gathered} \right) \hfill \\ \end{gathered} \right]} \right\} \\ & \quad - \frac{{P\left( {\text{1} + v} \right)}}{E}\text{Im}\left[ {C_{\text{1}} + \frac{{\left( \begin{gathered} C_{\text{0}} + C_{\text{1}} \zeta + C_{\text{2}} \zeta^{{\text{ - 1}}} + C_{\text{3}} \zeta^{{\text{ - 2}}} \hfill \\ + C_{\text{4}} \zeta^{{\text{ - 3}}} + C_{\text{5}} \zeta^{{\text{ - 4}}} + C_{\text{6}} \zeta^{{\text{ - 5}}} \hfill \\ + C_{\text{7}} \zeta^{{\text{ - 6}}} + C_{\text{8}} \zeta^{{\text{ - 7}}} + C_{\text{9}} \zeta^{{\text{ - 8}}} \hfill \\ \end{gathered} \right)}}{{\text{2}\left( \begin{gathered} C_{\text{1}} \zeta - C_{\text{2}} \zeta^{{ - \text{1}}} - \text{2}C_{\text{3}} \zeta^{{ - \text{2}}} \hfill \\ - \text{3}C_{\text{4}} \zeta^{{ - \text{3}}} - \text{42}C_{\text{5}} \zeta^{{ - \text{4}}} - \text{5}C_{\text{6}} \zeta^{{ - \text{5}}} \hfill \\ - \text{6}C_{\text{7}} \zeta^{{ - \text{6}}} - \text{7}C_{\text{8}} \zeta^{{ - \text{7}}} - \text{8}C_{\text{9}} \zeta^{{ - \text{8}}} \hfill \\ \end{gathered} \right)}} \times \left( \begin{gathered} C_{\text{1}} + C_{\text{2}} \zeta^{\text{2}} + \text{2}C_{\text{3}} \zeta^{\text{3}} \hfill \\ + \text{3}C_{\text{4}} \zeta^{\text{4}} + \text{4}C_{\text{5}} \zeta^{\text{5}} \hfill \\ + \text{5}C_{\text{6}} \zeta^{\text{6}} + \text{6}C_{\text{7}} \zeta^{\text{7}} \hfill \\ + \text{7}C_{\text{8}} \zeta^{\text{8}} + \text{8}C_{\text{9}} \zeta^{\text{9}} \hfill \\ \end{gathered} \right)} \right]. \\ \\ \end{aligned}$$

In order to consider the influence of the inclination angle, in the mapping process, the section of the roadway is rotated counterclockwise by an angle α (the inclination angle of the coal seam), then the polar coordinate transformation is *ζ* = *ρ*(cos(*θ* + *α*) + *i*sin(*θ* + *α*)). Bring *ζ* = *ρ*(cos(*θ* + *α*) + *i*sin(*θ* + *α*)) and *C*_*j*_ = *a*_*j*_ + *id*_*j*_ into (26) and (29), use Matlab software to separate the real and imaginary parts, you can get analytical solution of stress *σ*_*θ*_ and displacement *u*_*ρ*_ of inclined coal seam roadway.

### Example analysis of roadway

#### Roadway calculation azimuth layout

**Figure 7:**
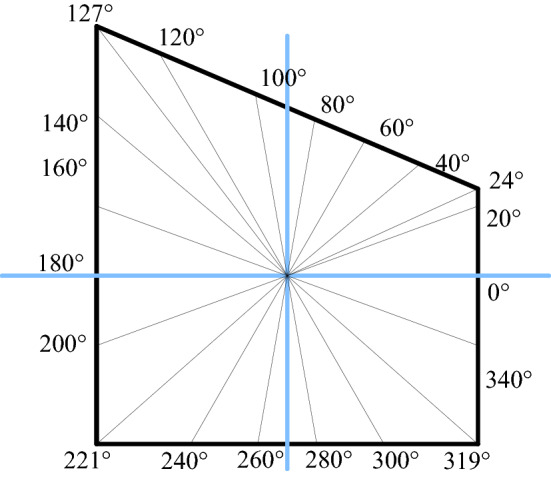
Roadway calculation azimuth layout.

The inclination angle of the inclined coal seam in Shitanjing No. 2 mining area is 18°–27°, with an average of 23°. Take the overlying rock layer pressure (in-situ stress) *P* as 10 MPa, assuming that the rock layer is homogeneous, its elastic modulus is 3.5 GPa, and Poisson's ratio is 0.24. Substituting the coefficients of the mapping function in Table 1 into Eqs. (26) and (27) respectively, the surface hoop stress and radial displacement of the roadway can be obtained. The layout of the calculation points for the stress and displacement of the roadway surrounding rock is shown in Fig. 7.

#### Stress distribution characteristics of roadway surrounding rock

**Figure 8:**
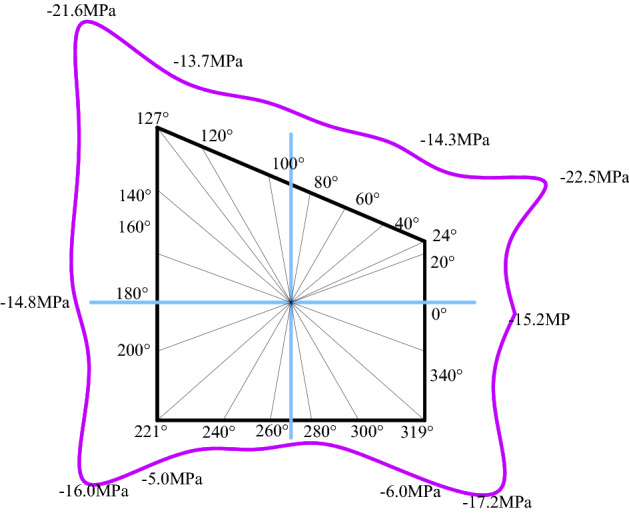
Stress distribution law of roadway surrounding rock.

Figure 8 shows the stress distribution of roadway surrounding rock. It can be seen from the figure that the peak stress on the right side of the roadway (sharp corner, two sides, roof, and floor) is greater than that on the left, and the peak stress at the two sharp corners of the roadway roof is greater than that at the two sharp corners of the floor. Overall, the stress of roadway surrounding rock shows the changing trend of sharp angle > two sides > roof > floor. The maximum stresses of roadway right side roof angle, right side, left side, roof, and floor are 22.5 MPa, 15.2 MPa, 14.8 MPa, 14.3 MPa, and 7.0 MPa, respectively.

#### Deformation distribution characteristics of roadway surrounding rock

**Figure 9:**
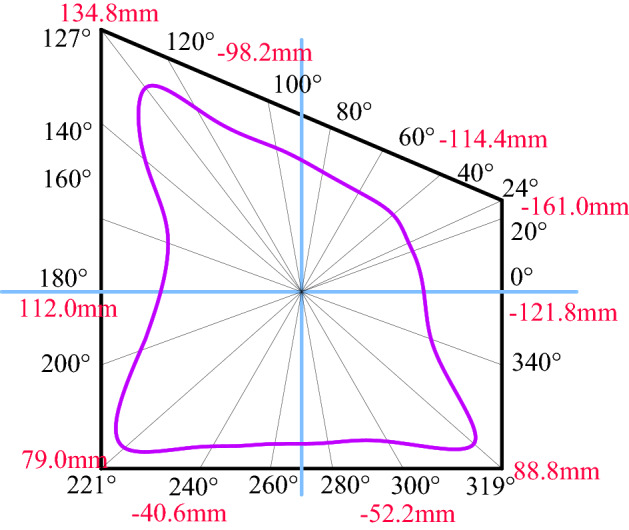
Deformation law of roadway surrounding rock.

Figure 9 shows the displacement distribution of roadway surrounding rock. It can be seen from the figure that the deformation of roadway surrounding rock presents asymmetric distribution characteristics, that is, the deformation on the right side of roadway sharp corner, two sides and roof, and the floor is greater than that on the left. On the whole, the deformation at the top corner of the right side of the roadway is the largest, followed by the two sides of the roadway, and the smallest is the roadway roof and floor. The displacements of the top corner of the right side of the roadway, the left side, the right side, the roof, and the floor are 161.0 mm, 112.0 mm, 121.8 mm, 114.4 mm, and 52.5 mm, respectively.

### Theory of cyclic deformation and failure of roadway surrounding rock

#### Shape of roadway stress and deformation zone

**Figure 10:**
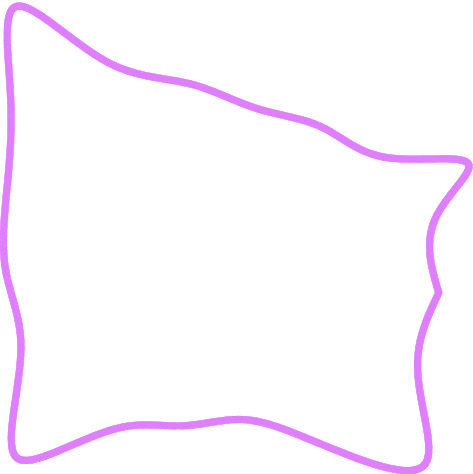
Boundary of stress zone.

**Figure 11:**
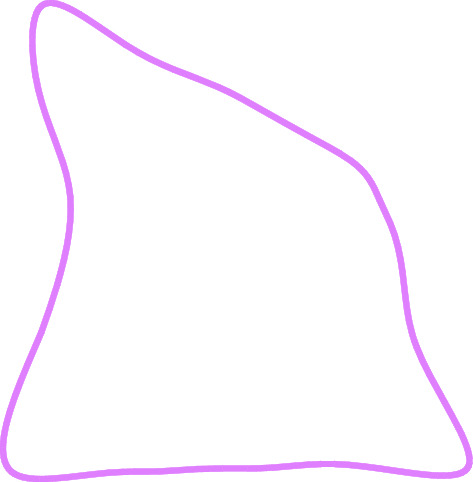
Boundary of deformation zone.

**Figure 12:**
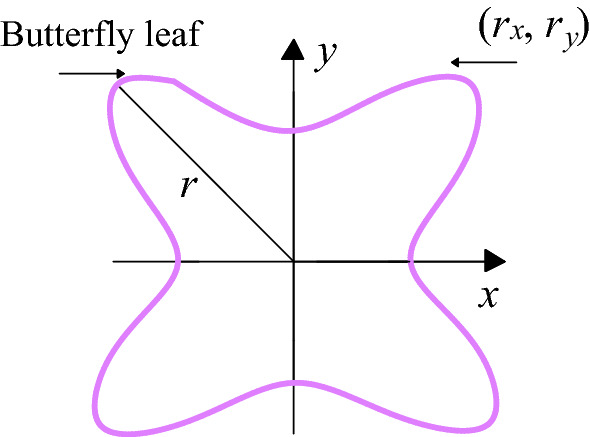
Meaning of butterfly boundary.

The contour of stress and deformation of surrounding rock of roadway in inclined coal seam presents a butterfly shape, and the protruding part is called butterfly leaf. As shown in Figs. 10, 11 and 12, in the coordinate system, it presents the shape of mutual four quadrant protruding and coordinate axi + s depression, in which *r*_*D*_ is the length of butterfly leaf, (*r*_*x*_*,r*_*y*_) is the coordinate of any point on the stress area and deformation boundary of roadway surrounding rock, with |*r*_*D*_| >|*r*_*y*_| >|*r*_*x*_|.

#### Cyclic deformation and failure characteristics of roadway surrounding rock

**Figure 13:**
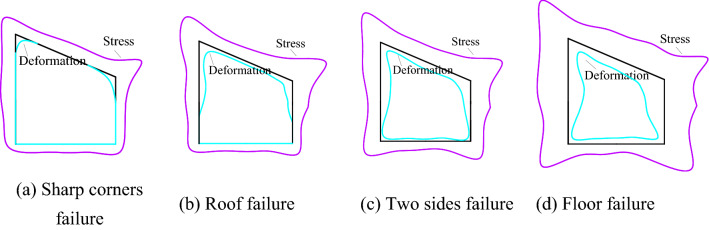
Cyclic deformation and failure of roadway surrounding rock.

As shown in Fig. 13, the stress concentration occurs at the butterfly leaf position in the stress area of the roadway surrounding rock, and the two sharp corners of the roadway roof are damaged. With the increase of the butterfly leaf in the stress area, the damage of the two sharp corners of the roof is intensified, increasing the roof span. It increases the stress of the two sides of the roadway, resulting in the bulging of the side of the roadway. With the continuous increase of stress, butterfly leaves in the stress area are generated at the two sharp corners of the roadway floor, and a slight floor heave occurs. Then the roof, floor, two sides, and sharp corners of the roadway surrounding rock interact, resulting in the increase of stress and the decrease of strength, and the roadway surrounding rock enters a vicious cycle of damage.

#### Establish the theory of cyclic deformation and failure of roadway

**Figure 14:**
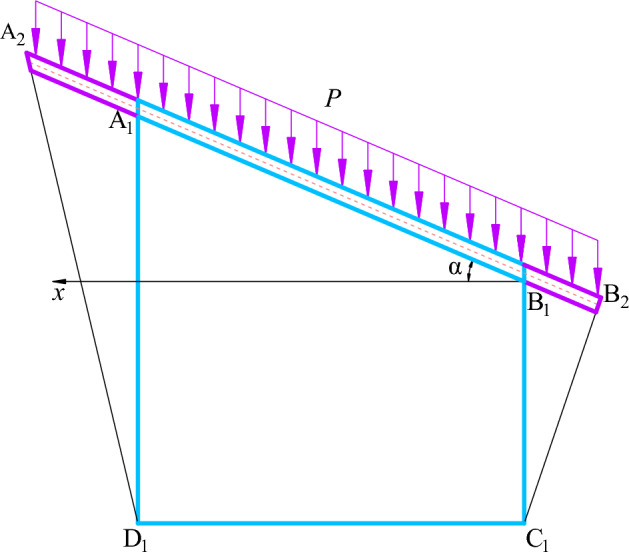
Mechanical model of roadway cyclic deformation and failure.

**Figure 15:**
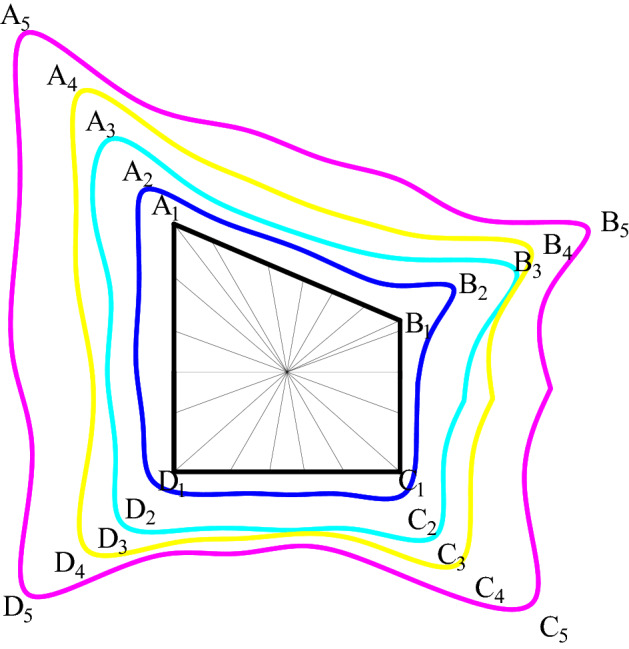
Stress distribution of roadway cyclic deformation failure.

Based on the theory of mathematics and elasticity, combined with the distribution form of stress and deformation of roadway surrounding rock, the theoretical expression of roadway surrounding rock cyclic deformation and failure is deduced, and the structural length of the roof, two sides and floor and the analytical solution of roadway stress and deformation in the whole life cycle of roadway surrounding rock cyclic deformation and failure can be calculated. Figures 14 and 15 show the mechanical model of cyclic deformation and failure of roadway surrounding rock. The length expression (27) of the roadway roof, two sides, and the floor are shown. According to Eqs. (25) and (26), the stress and deformation values of roadway roof, two sides, and floor can be obtained.

27 $$\left\{ \begin{gathered} L_{{A_{{\text{n}}} B_{n} }} = \sqrt {\text{(}x_{{A_{{\text{n}}} }} - x_{{B_{n} }} \text{)}^{2} + \text{(}y_{{A_{{\text{n}}} }} - y_{{B_{n} }} \text{)}^{2} } \hfill \\ L_{{B_{{\text{n}}} C_{n} }} = \sqrt {\text{(}x_{{B_{{\text{n}}} }} - x_{{C_{n} }} \text{)}^{2} + \text{(}y_{{B_{{\text{n}}} }} - y_{{C_{n} }} \text{)}^{2} } \hfill \\ L_{{C_{{\text{n}}} D_{n} }} = \sqrt {\text{(}x_{{C_{{\text{n}}} }} - x_{{D_{n} }} \text{)}^{2} + \text{(}y_{{C_{{\text{n}}} }} - y_{{D_{n} }} \text{)}^{2} } \hfill \\ L_{{D_{{\text{n}}} A_{n} }} = \sqrt {\text{(}x_{{D_{n} }} - x_{{A_{n} }} \text{)}^{2} + \text{(}y_{{D_{n} }} - y_{{A_{n} }} \text{)}^{2} } \hfill \\ \end{gathered} \right.\text{,n = 1,2,3,4,5} \cdots$$

Put *z* = *x* + *iy*, *ζ* = *ρ*(cos*θ* + *i*sin*θ*) = *ρe*^*iθ*^, *C*_*j*_ = *a*_*j*_ + *id*_*j*_, *ρ* = into Eq. (15), we can get::

28 $$\left\{ \begin{gathered} x_{i} = a_{\text{0}} + a_{\text{1}} \text{cos}\theta - d_{\text{1}} \text{sin}\theta + \sum\limits_{{j = \text{2}}}^{n} {\left[ {a_{j} \text{cos(}j - \text{1)}\theta + d_{j} \text{sin(}j - \text{1)}\theta } \right]} \hfill \\ y_{i} = d_{\text{0}} + a_{\text{1}} \text{sin}\theta + d_{\text{1}} \text{cos}\theta + \sum\limits_{{j = \text{2}}}^{n} {\left[ { - a_{j} \text{sin(}j - \text{1)}\theta + d_{j} \text{cos(}j - \text{1)}\theta } \right]} \hfill \\ \end{gathered} \right.,\quad i = \text{A}_{\text{n}} \text{,B}_{\text{n}} \text{,C}_{\text{n}} \text{,D}_{\text{n}} ,$$

where *L*_*AnBn*_, *L*_*BnCn*_, *L*_*CnDn*_ and *L*_*DnAn*_ are the lengths of the roof, sides and bottom of the roadway, respectively. The angle *θ* is 0°–360°, which are the measuring points for the stress and deformation of the roadway. The abscissas of A, B, C, D around the roadway are *x*_*An*_, *x*_*Bn*_, *x*_*Cn*_, *x*_*Dn*_, and the ordinates are *y*_*An*_, *y*_*Bn*_, *y*_*Cn*_, *y*_*Dn*_, n = 1,2,3,4,5….

The roadway section is mapped to the unit circle through conformal transformation, and the mapping function coefficients are unchanged at this time. With the increase of θ, the values of cos*θ* and sin*θ* in the equation (2.58) change. When *θ* increases near *k*π/2 + π/4 (*k* = 0, 1, 2, 3), the boundary of the stress zone and the deformation zone presents a butterfly-shaped contour. The larger position of the butterfly leaf in the butterfly stress zone indicates that the sharp corner of the roadway roof is the source of damage, which is prone to stress concentration. As the load increases, the two sides of the roadway are damaged, which leads to the increase of the roof span *L*_*AnBn*_ and the stress of the roof. At the same time, the damage of the roof increases the length of the two sides *L*_*BnCn*_ and *L*_*CnDn*_, which intensifies the damage of the two sides, resulting in the continued deterioration of the stress state of the roadway sharp corners and aggravating the roof sinking. The roof span *L*_*AnBn*_ is further increased, and the damage of the two sides of the roadway extends to the deep part, which deteriorates the stress condition of the bottom plate, and causes the span of the bottom plate *L*_*DnAn*_ to increase, which enters a vicious circle of destruction.

## Numerical simulation analysis of roadway deformation and failure

### Numerical calculation model

**Figure 16:**
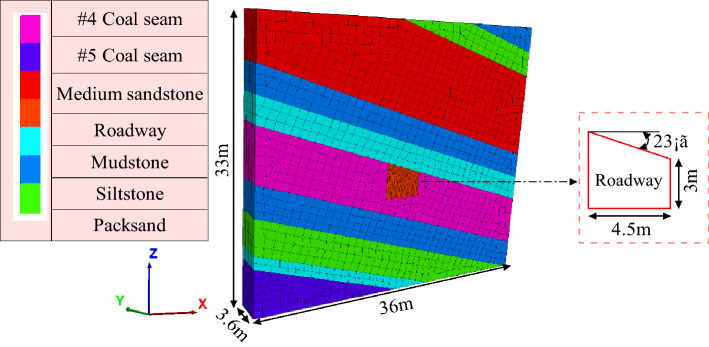
Model.

Taking Shitanjing No. 2 mining area as the engineering background, the FLAC3D numerical calculation model is established, with a size of 36 m × 3.6 m × 33 m and the coal seam dip angle is 23°. The grid is divided into 13,381 units, focusing on the grid around the roadway^25^, as shown in Fig. 16. Since the buried depth is about 400 m, 10 MPa stress is applied on the top surface of the model. The parameters of the numerical calculation model are shown in Table 2.

**Table 2:** Physical and mechanical parameters of coal and rock.

Rock stratum	Thickness/m	Density/g cm^3^	Bulk modulus /GPa	Shear modulus/GPa	Friction/°	Cohesion /MPa	Tension /MPa
#3 Coal seam	3.15	1.40	2.00	1.10	26.0	1.20	1.00
Siltstone	3.00	2.46	8.49	6.47	32.1	5.7	3.77
Packsand	3.00	2.5	8.24	5.92	30.16	9.62	2.27
Medium sandstone	10.00	2.51	10.11	7.27	37.0	11.8	2.78
Siltstone	3.00	2.46	8.49	6.47	32.1	5.7	3.77
Mudstone	3.00	2.53	7.79	5.34	31.5	1.85	1.54
#4 Coal seam	6.00	1.40	2.00	1.10	28.0	1.40	1.00
Siltstone	3.50	2.46	8.49	6.47	32.1	5.7	3.77
Packsand	4.00	2.5	8.24	5.92	30.16	9.62	2.27
Mudstone	1.50	2.53	7.79	5.34	31.5	1.85	1.54
#5 Coal seam	5.5	1.40	2.00	1.10	26.0	1.20	1.00

### Result analysis

#### Stress distribution characteristics of roadway

**Figure 17:**
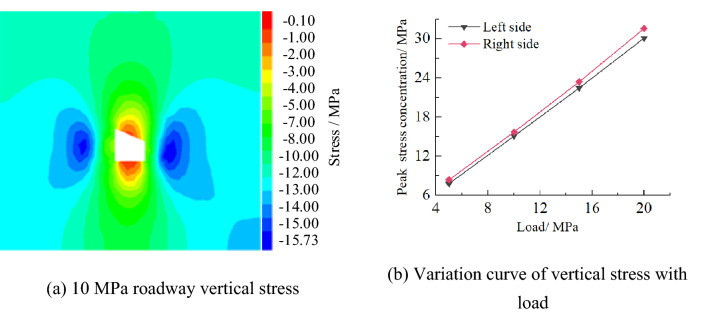
Vertical stress distribution law of roadway.

1. Vertical stress
  Figure 17 shows the vertical stress distribution law of roadway in inclined coal seam. It can be seen from the figure that the vertical stress concentration peak of roadway appears in the side of roadway and presents asymmetric distribution characteristics, that is, the range of stress peak and stress concentration area of roadway on the right side is larger than that on the left side, and the stress distribution of roadway roof and floor deviates to the right. With the increase of load, the stress of two sides of roadway increases. When the load is 10 MPa, the peak stress of the left and right sides of the inclined coal seam roadway is 15.20 MPa and 15.73 MPa respectively.

(2) Horizontal stress

**Figure 18:**
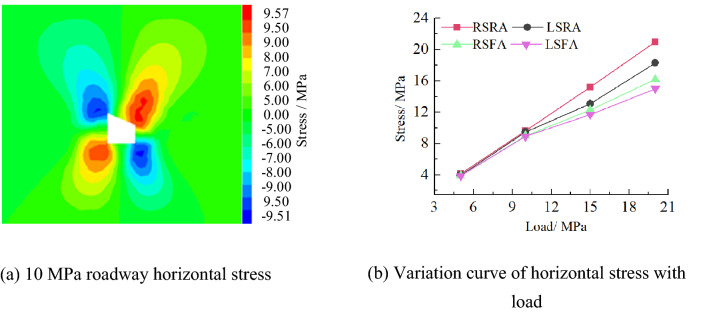
Horizontal stress distribution law of roadway.

Figure 18 shows the horizontal stress distribution law of roadway surrounding rock in inclined coal seam. From the figure, it can be seen that the horizontal stress concentration of roadway surrounding rock occurs at four sharp corners and presents asymmetric "Butterfly" distribution characteristics, that is, the range of stress peak and stress peak area at the sharp corner on the right side of the roadway is greater than that on the left side. With the increase of load, the stress at the sharp corner of the roadway increases. When the load is 10 MPa, the peak stress of the right side roof angle (RSRA), the left side roof angle (LSRA), the right side floor angle (RSFA) and the left side floor angle (LSFA) of the roadway are 9.57 MPa, 9.51 MPa, 9.03 MPa and 8.94 MPa respectively.

#### Distribution characteristics of roadway displacement

**Figure 19:**
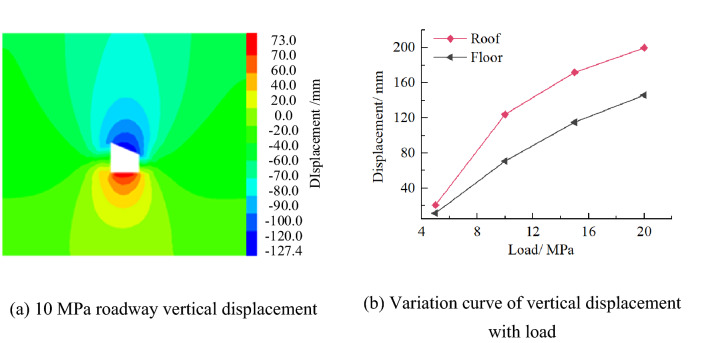
Distribution law of roadway vertical displacement.

1. Vertical displacement
  Figure 19 shows the vertical displacement distribution law of roadway. It can be seen from the figure that the deformation of roadway roof and floor is skewed on the right side, showing asymmetric distribution characteristics. The right side of roadway roof and floor is the key part of roadway deformation. With the increase of load, the deformation of roadway roof and floor is greater. When the load is 10 MPa, the maximum displacement of roadway roof and floor is 127.4 and 73.0 mm, respectively.

(2) Horizontal displacement

**Figure 20:**
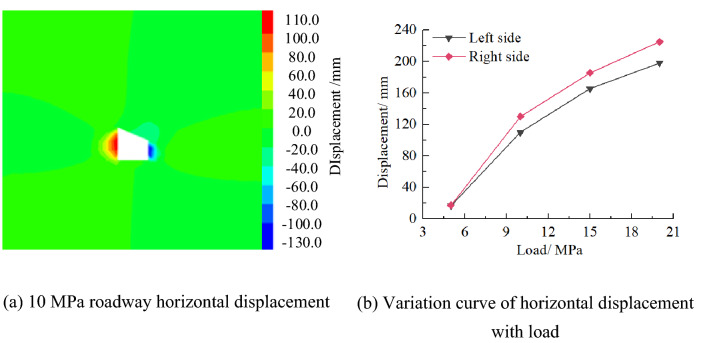
Distribution law of roadway horizontal displacement.

Figure 20 shows the horizontal displacement nephogram of the roadway. It can be seen from the figure that the deformation of two sides of the roadway presents asymmetric characteristics, that is, the deformation of the right side of the roadway is greater than that of the left side. With the increase of load, the deformation of two sides of roadway is greater. When the load is 10 MPa, the maximum deformation of the left and right sides of the inclined coal seam roadway is 16.8 and 17.3 mm, respectively.

#### Distribution characteristics of roadway plastic zone

**Figure 21:**
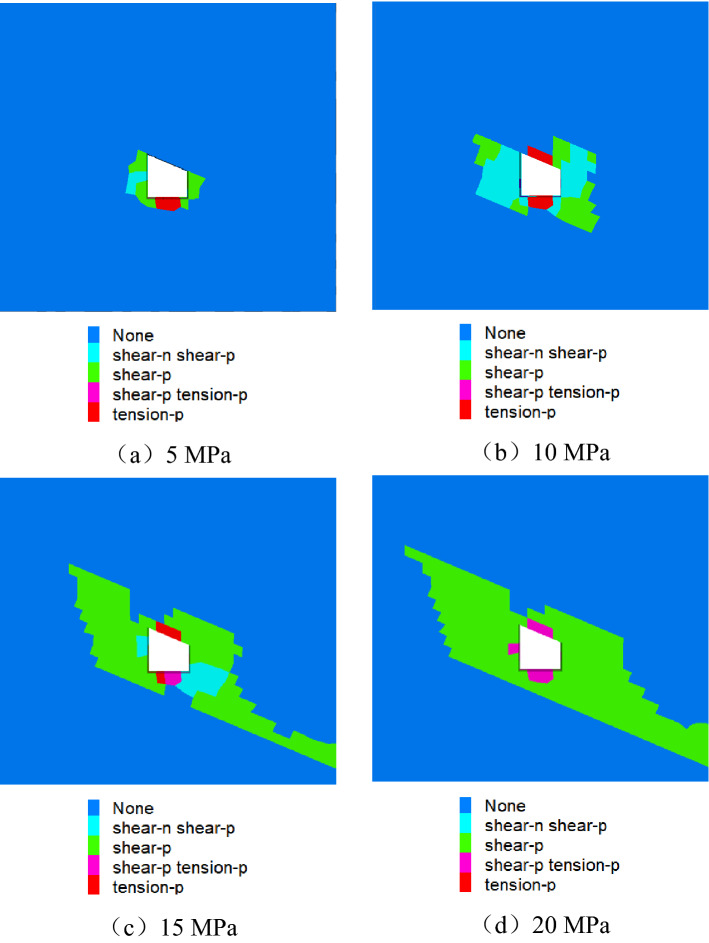
Roadway plastic zone.

Figure 21 shows the cloud diagram of the plastic area of the roadway under different loads. It can be seen from the figure that the plastic area of the roadway is distributed in an asymmetric "Butterfly" along the inclined direction of the coal seam. Under the action of low load, the roadway plastic zone appears in the sharp corner and side of the roadway. With the increase of load, the plastic zone at the sharp corner of the roadway first expands to the roof, floor and the right side roof angle of the roadway. The interaction of various parts of the roadway leads to increased roadway stress, decreased strength, and finally asymmetric cyclic deformation and failure.

### Comparative analysis of theoretical calculation and numerical simulation results

**Figure 22:**
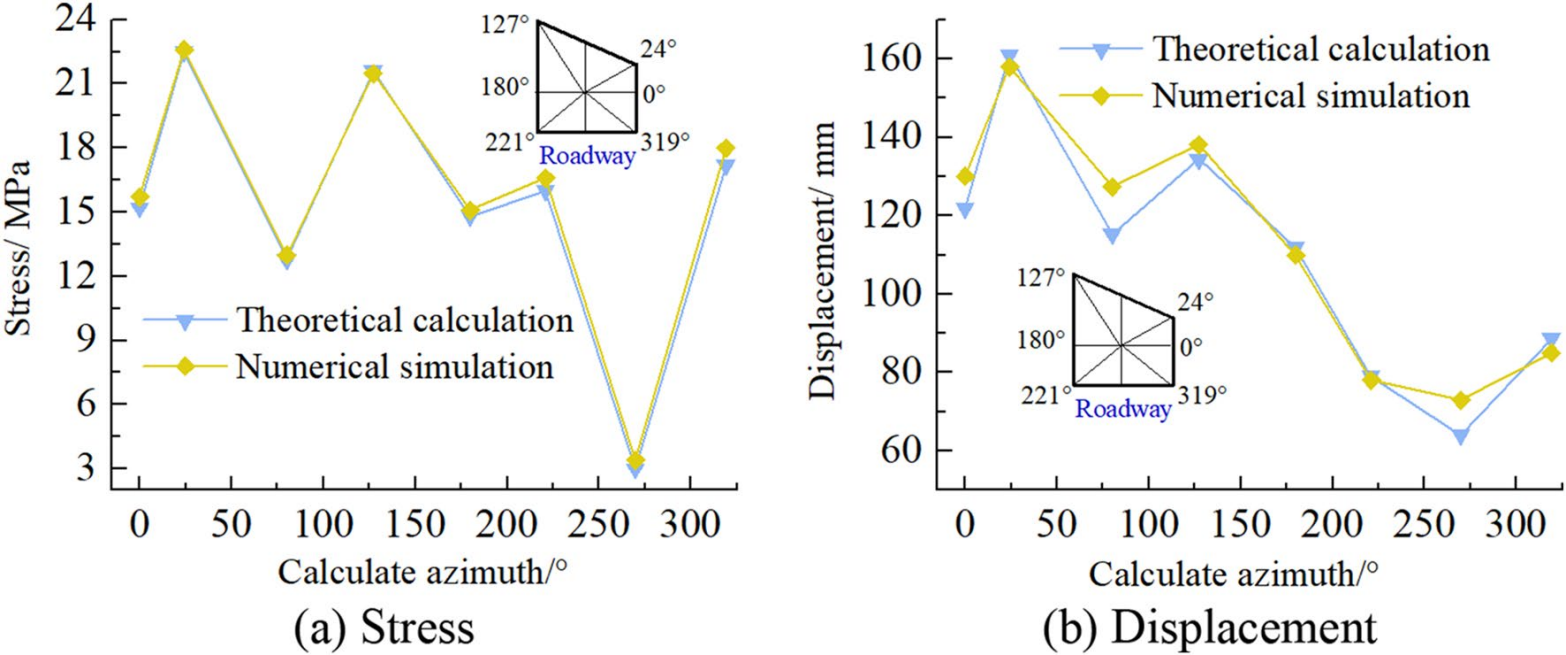
Comparison between theoretical calculation and numerical simulation results.

To verify the rationality and accuracy of theoretical calculation, the stress and displacement calculated by numerical simulation are converted into circumferential stress and radial displacement, compared with theoretical analysis and calculation results. As shown in Fig. 22, the theoretical analysis shows that the distribution law of roadway stress and displacement is consistent with the numerical simulation. There are some numerical differences, and the maximum numerical difference is less than 10%. This may be related to the calculation process of the two algorithms, in which the theoretical analysis is based on elasticity. In contrast, the numerical simulation analysis adopts the elastic–plastic constitutive model. It is also related to the size of computational boundary conditions and the difference of mesh generation of a numerical model. Therefore, there are some differences between theoretical calculation and numerical simulation results.

## Experimental study on deformation and failure mechanism of roadway

### Physical simulation test scheme

**Figure 23:**
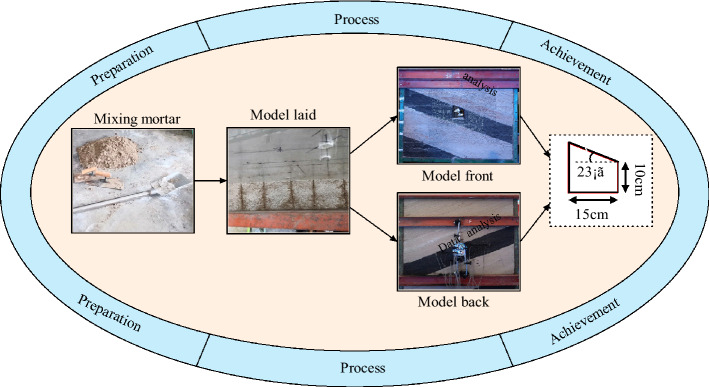
Physical simulation test system.

Taking the engineering geological conditions of Shitanjing No. 2 mining area as the background (the physical and mechanical parameters of coal seams are shown in Table 1), a variable-angle physical model test frame is used to establish a physical similarity model for inclined coal seam roadways, as shown in Fig. 23. The establishment of the similarity model meets the conditions of geometric, bulk density, material and stress similarity, and the size of the roadway model shall meet the requirement that the ratio of the distance from the roadway to the model boundary to the radius of the roadway is ≥ 3. The similarity parameters of the physical model are shown in Table 3. Similar materials are mainly composed of sand, gypsum, CaCO_3_ and water in a certain proportion. The model consists of 10 layers, which are stacked in layers. The ratio of similar materials is shown in Table 4.

**Table 3:** Similar parameters of physical model.

Item	Parameter (m)	Item	Parameter
Model length	1.2	Geometric similarity ratio	1:30
Model width	0.12	Bulk density similarity ratio	1:1.7
Model height	1.1	Stress similarity ratio	1:51
Model boundary	0.525	Load similarity ratio	1:51

**Table 4:** Ratio of physical similar material simulation test.

Layer number	Kinds of strata	Layer thickness/cm	Ratio (sand:gypsum:CaCO_3_:coal)
1	Siltstone	10	737
2	Packsand	10	837
3	Medium sandstone	33	728
4	Siltstone	10	737
5	Mudstone	10	828
6	#4 Coal seam	20	21:1:2:21
7	Siltstone	12	737
8	Packsand	13	837
9	Mudstone	5	828
10	5 #Coal seam	18	21:1:2:21

Note: 737 proportion number. The first number 7 indicates that the sand binder ratio is 7:1, and the second and third numbers 3 and 7 indicate that the ratio of gypsum to large lime is 3:7.

In this study, two hydraulic jacks are used to apply uniform load on the top surface of the physical model. Starting from 0.021 Mpa, execute the graded loading method, as shown in Table 5.

**Table 5:** Loading scheme.

Load times	1	2	3	4	5	6	7	8	9	10	11	12	13
Load/kN	3	4	5	6	7	8	9	10	11	12	13	14	15
Load/MPa	0.021	0.028	0.035	0.042	0.049	0.063	0.07	0.077	0.084	0.091	0.098	0.105	0.112

### Physical simulation test process

**Figure 24:**
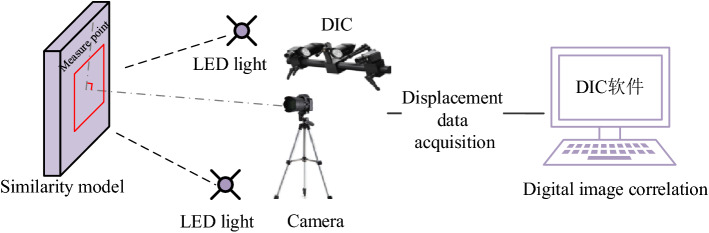
DIC measurement system.

Miniature earth pressure sensor (L-YB-150) is adopted (φ 28 mm × 9 mm, 0.5 (% F.S.) and DH3818-1 static strain tester were used to record the stress changes, and DIC was used to record the displacement and failure law of roadway. Figure 24 shows the DIC test system. Its image consists of two sets with a resolution of 2648 × 2448. The sub-pixel accuracy of pixel CCD digital camera acquisition can be realized by using 3D-DIC software (GOM Aramis, version 8, related solutions). The shooting format of this experiment is 1.2 × 1.1 m, the magnification is 3.383 pixels/mm, and the DIC displacement measurement accuracy is expected to be 0.0296 mm^26–33^. Compared with traditional monitoring methods, DIC test system has short monitoring time intervals and high precision. It can realize high-precision testing of large-scale and detailed parts of rock stratum at the same time, and master the dynamic process of rock stratum deformation and failure.

**Figure 25:**
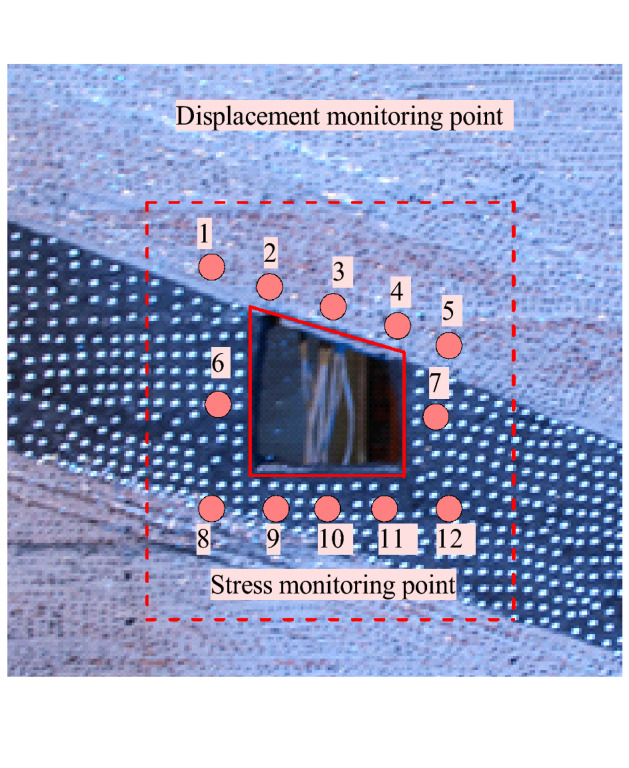
Layout of stress and displacement measuring points of roadway.

Based on the deformation and failure of inclined coal seam roadway, the stress and displacement monitoring points are arranged, as shown in Fig. 25. Three stress monitoring points are arranged on the roadway roof, two side walls, floor and four sharp corners respectively. A total of 12 stress sensors are embedded in the roadway surrounding rock, and the measurement error is 0.5%. Displacement monitoring points are arranged in the influence range of roadway surrounding rock surface deformation for monitoring by DIC. At the same time, in order to eliminate the rigid body displacement, eight fixed control marks are placed on the physical model frame to correct the DIC measurement error. After the model is cured for one month to reach the expected mechanical strength and its water content meets 1.6–2.7%, the loading test can be carried out^34–38^. During the loading process, the stress, deformation and failure characteristics of two sides, roof, floor and sharp corners are recorded.

### Results and interpretation

#### Stress analysis

1. Stress of roadway sidewall

**Figure 26:**
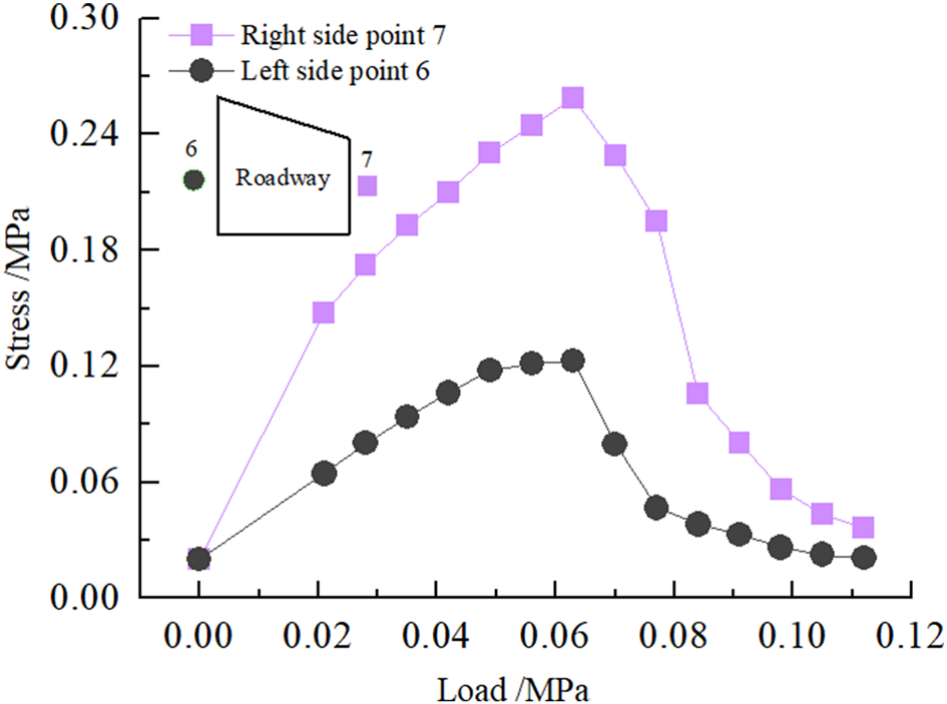
Curve of stress on roadway's two sidewalls with load.

Figure 26 shows the stress distribution law of the roadway's two sides. It can be seen from the figure that in the loading stage of 0–0.063 MPa, the stress of two sides of the roadway increases rapidly with the increase of load, resulting in stress concentration. The stress concentration on the two sides reaches the maximum when the load is 0.063 MPa, and the stress concentration factors on the left and right sides are 2.0 and 4.1 respectively. At this time, cracks appear on two sides of the roadway, with slight wall spalling. In the loading stage of 0.063–0.112 MPa, the stress of the two sides of the roadway decreases rapidly, indicating that the roadway's two sides have been greatly damaged. There are large cracks on the inner side of the two sides of the roadway, and the two sides bulge seriously. The damage to the right side of the roadway is greater than that of the left side. The peak stress of the left and right sides of the roadway are 0.12 and 0.24 MPa, respectively.

(2) Stress of roadway roof

**Figure 27:**
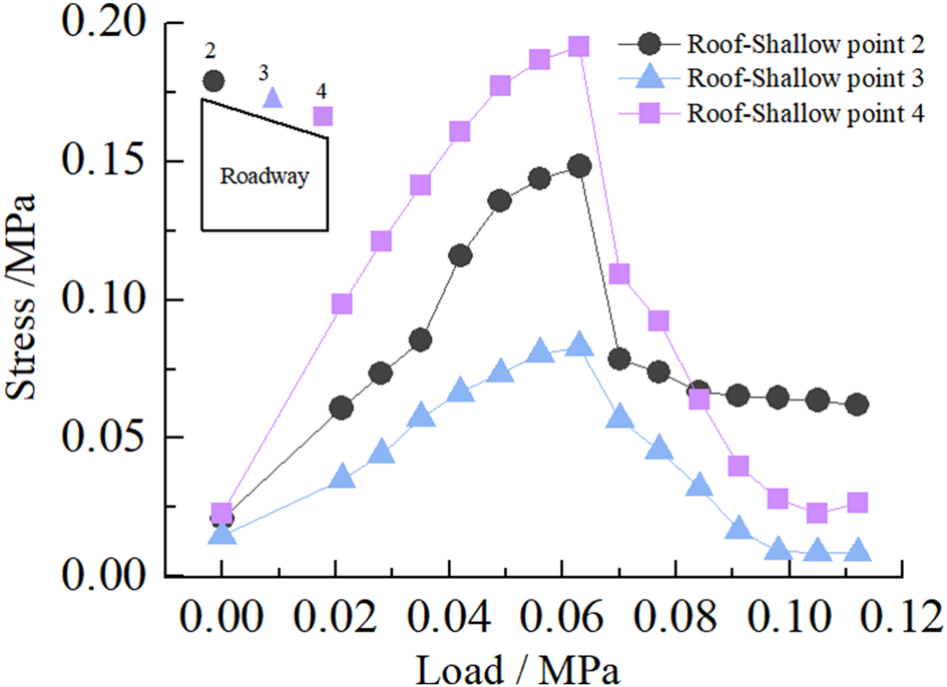
Curve of roadway's roof stress with load.

Figure 27 shows the stress distribution law of roadway roof surrounding rock. In the loading stage of 0–0.063 MPa, the stress of the roadway roof increases with the increase of load, and stress concentration occurs. When the load reaches 0.063 MPa, the stress concentration on the left and right sides of the roadway roof reaches the maximum, and the stress concentration factors are 2.8 and 3.6, respectively. At this time, the roadway roof separates and local collapse occurs. In the loading stage of 0.063–0.112 MPa, the stress of the roadway roof decreases rapidly. At this time, the roadway roof collapses, the deformation of the right side of the roof is greater than that of the left side, and the stress peaks on the left and right sides of the roof are 0.15 and 0.19 MPa, respectively.

(3) Stress distribution of roadway floor

**Figure 28:**
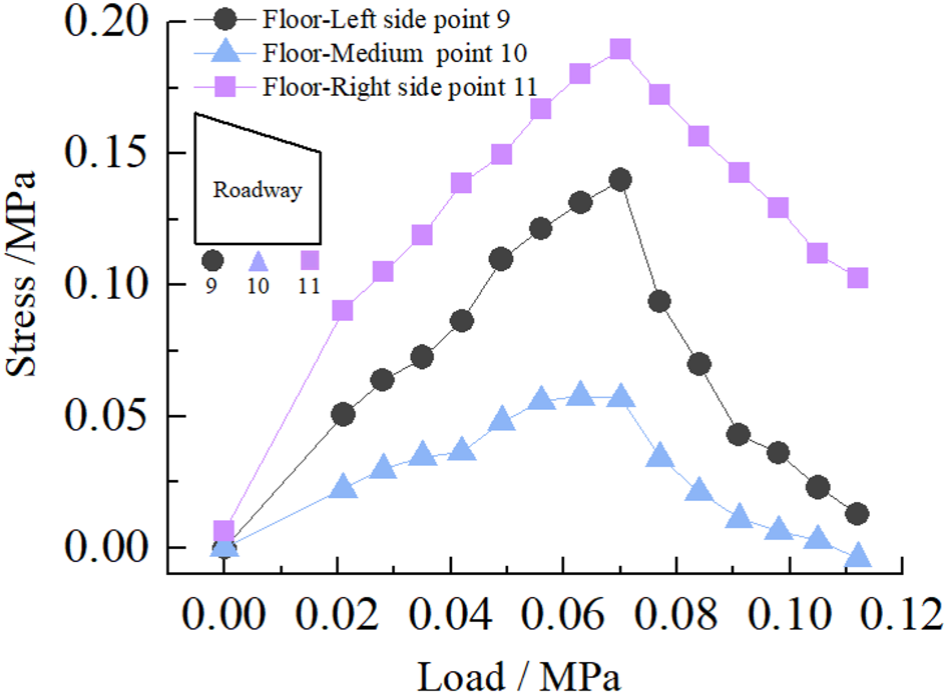
Curve of roadway floor stress with load.

Figure 28 shows the stress distribution law of the surrounding rock of roadway floor. It can be seen from the figure that in the loading stage of 0–0.063 MPa, the surrounding rock stress of roadway floor increases rapidly with loading, and stress concentration occurs. When the load reaches 0.063 MPa, the stress concentration on the left and right sides of the roadway floor reaches the maximum, and the stress concentration factors are 2.0 and 2.7, respectively. At this time, the roadway floor begins to crack. In the loading stage of 0.063–0.112 MPa, the surrounding rock stress of the roadway floor decreases rapidly with the increase of load. At this time, the crack of the roadway floor extends to the interior of the roadway, there is a slight floor heave, and the deformation on the right side of the floor is greater than that on the left side. The peak stress of the two surrounding rocks on the left and right sides of the roadway floor is 0.14 and 0.185 MPa, respectively.

(4) Stress distribution at corner of roadway

**Figure 29:**
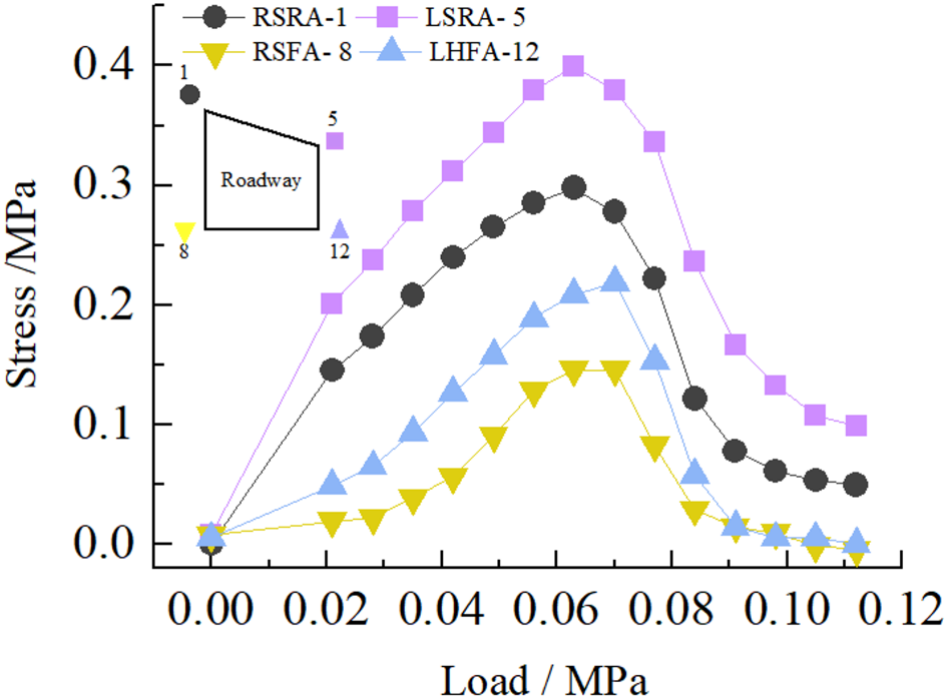
Curve of stress at sharp corner of roadway with load.

Figure 29 shows the stress distribution law of surrounding rock at the sharp corner of the roadway. It can be seen from the figure that in the loading stage of 0–0.063 MPa, the stress of the surrounding rock of the roadway increases rapidly with the loading, resulting in stress concentration. When the load reaches 0.063 MPa, the stress concentration at the sharp corner of the roadway reaches the maximum, and the stress concentration factors at the sharp corner are 5.4, 4.0, 3.2, and 2.1, respectively. At this time, cracks begin to appear at the sharp corner of the roadway. In the loading stage of 0.063–0.112 MPa, the stress of surrounding rock at the sharp corner of the roadway decreases rapidly with the increase of load. At this time, the cracks at the sharp corner of the roadway increase, and finally, the surrounding rock at the sharp corner of the roadway collapses, and the damage of the sharp corner of the right side of the roadway is greater than that of the left side. The peak stress of the right side roof angle (RSRA), the left side roof angle (LSRA), the right side floor angle (RSFA), and the left side floor angle (LSFA) of the roadway are 0.38 MPa, 0.28 MPa, 0.22 MPa, and 0.16 MPa respectively.

#### Displacement of roadway surface

1. Surface displacement of two sidewalls of roadway

**Figure 30:**
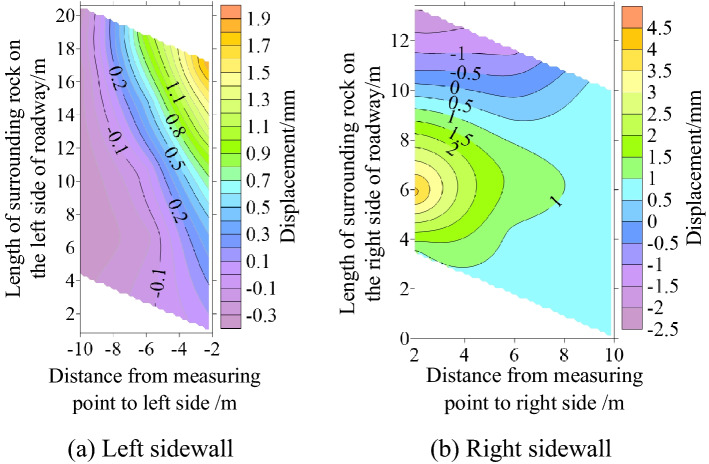
Displacement nephogram of all measuring points on both sides of roadway at 0.063 MPa.

Figure 30 shows the displacement nephogram of each measuring point on the two sides of the roadway. It can be seen from the figure that the displacement of the right side of the roadway is greater than that of the left side, showing asymmetric characteristics, in which the maximum displacement of the right side of the roadway is 2.10 mm and the maximum displacement of the left side is 1.93 mm. The deep surrounding rock deformation trend of the two sides of the roadway is consistent with the shallow part. With the increase of the depth of the surrounding rock of the two sides of the roadway, the deformation of the two sides is smaller.

(2) Roof displacement of roadway

**Figure 31:**
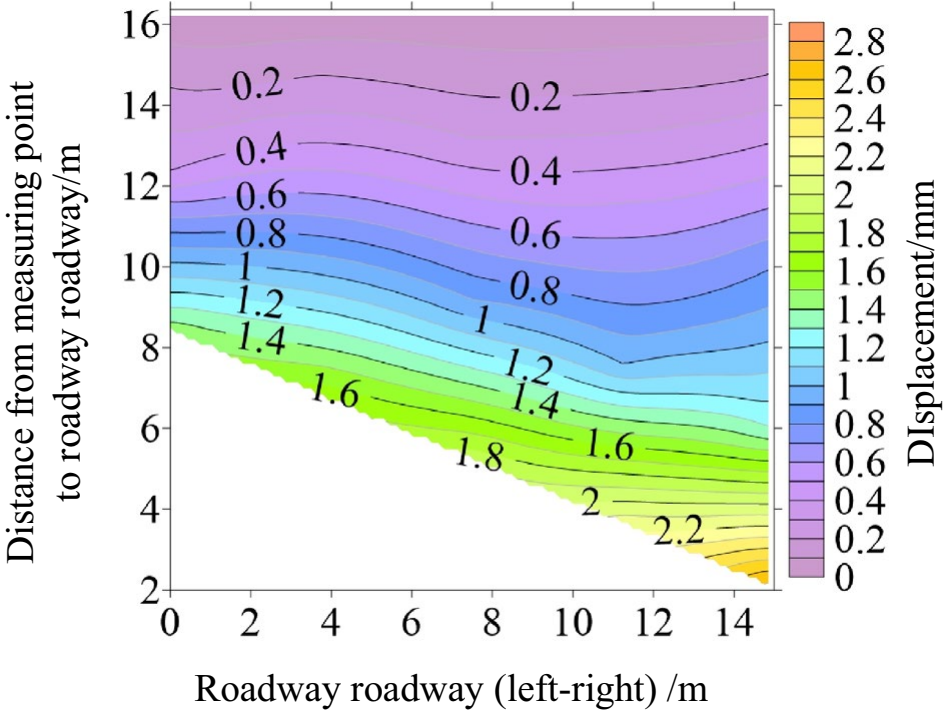
Displacement nephogram on roof of roadway at 0.063 MPa.

Figure 31 shows the displacement nephogram of each measuring point of roadway roof deformation. It can be seen from the figure that the displacement on the right side of the roof is greater than that on the left, showing asymmetric distribution characteristics, in which the maximum displacement of roadway roof is 2.77 mm. The deformation trend of deep surrounding rock of roadway roof is basically consistent with that of the shallow part. With the increase of surrounding rock depth of roadway roof, the deformation of roadway roof is smaller.

(3) Displacement of roadway floor

**Figure 32:**
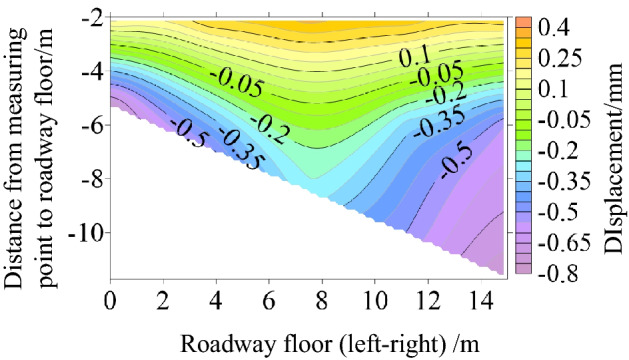
Displacement nephogram in roadway floor.

Figure 32 shows the displacement nephogram of each measuring point of the roadway floor. The deformation of the right side of the roadway floor is greater than that of the left side, showing asymmetric distribution characteristics, in which the maximum displacement of the roadway floor is 0.71 mm. The deformation trend of deep surrounding rock of roadway floor is basically consistent with that of shallow part. With the increase of surrounding rock depth of roadway floor, the deformation of roadway floor is smaller.

### Cyclic deformation and failure of roadway surrounding rock

**Figure 33:**
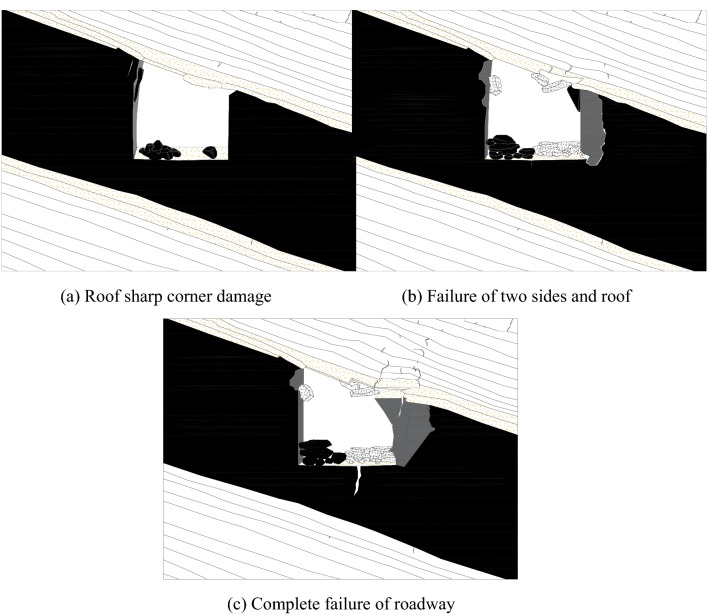
Cyclic deformation and failure of roadway surrounding rock.

Under the load action, the inclined coal seam roadway has cyclic deformation and failure, showing asymmetric characteristics. Stress concentration is easy to appear at the sharp corner of the roadway roof. With the increase of load, the stress concentration of roadway roof, two sides, and floor also reach the maximum. At this time, cracks appear in the roof, two sides, and floor. With the increasing load, the roof slightly separates from the layer. The stress state at the sharp corner continues to deteriorate as a connecting part, intensifying the roadway's deformation and failure. The interaction between the two sides of the roadway, the roof, and the two sharp corners of the roof leads to the increase of roadway stress, the decrease of strength, and the entry into a vicious circle of damage. Finally, the two sides of the roadway are seriously divided, the roof presents asymmetric "Beret" type caving arch damage, and the floor heaves slightly, as shown in Fig. 33.

### Comparative analysis of theoretical calculation and experimental results

**Figure 34:**
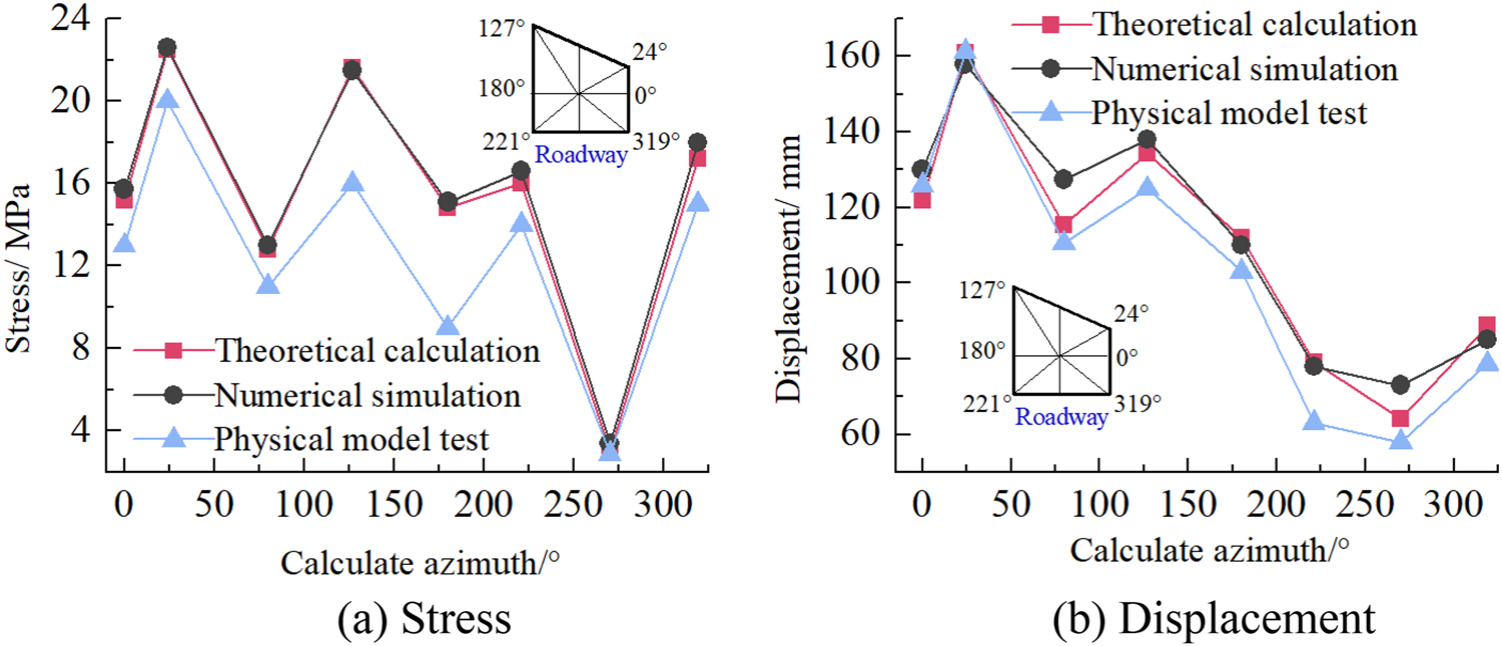
Comparison between physical test and theoretical calculation results.

To verify the rationality and accuracy of the theoretical calculation, the physical test results are converted by geometric similarity ratio and stress similarity ratio and compared with the theoretical calculation results. As shown in Fig. 34, the theoretical analysis shows that the distribution law of roadway stress and displacement is basically consistent with the physical test results. There are some differences in values, and the maximum difference is less than 32%. Because the results of theoretical analysis are elastic solutions based on complex function, while the results of numerical simulation are elastic–plastic solutions, the results of model tests are related to similar materials, the accuracy of test methods and test conditions, and the roadway is deformed and damaged. Therefore, some data are different.

## Conclusion

Based on the theory of complex function and elasticity, this paper establishes the mechanical model of roadway surrounding rock in inclined coal seam under the influence of dip angle, optimizes the solution process and accuracy of mapping function coefficient, and deduces the calculation equation of roadway surrounding rock stress and deformation. It reveals the deformation and failure mechanism of roadway in inclined coal seam, and is verified by numerical simulation and physical similarity simulation. The main conclusions are as follows:

1. The stress and deformation of roadway surrounding rock in inclined coal seam show obvious asymmetric distribution characteristics, that is, the stress and deformation on the right side of roadway surrounding rock are greater than that on the left side. On the whole, the stress and deformation of roadway surrounding rock show the change trend of side > roof > floor, and the two sides of roadway, the right side of roof and the top angle of right side are the key positions of stress concentration and deformation. The evolution mechanism of cyclic deformation and failure of roadway surrounding rock is further revealed, and the essential relationship between roadway roof and floor and deformation on both sides is established.
2. Using the numerical simulation analysis method, the stress, displacement and plastic zone distribution law of roadway surrounding rock in inclined coal seam are further analyzed. The roadway stress distribution and deformation law are basically consistent with the theoretical analysis results, and the numerical difference is less than 10%. The cyclic failure mechanism of inclined coal seam is verified. The stress concentration and deformation of surrounding rock in inclined coal seam roadway show asymmetric characteristics, that is, the stress and deformation on the right side are greater than that on the left side. The plastic zone of the roadway is asymmetrically "Butterfly" distributed along the inclined direction of the coal seam, that is, the range of the plastic zone on the right is greater than that on the left.
3. Through the physical model test, the stress and deformation law of the surrounding rock of the roadway in inclined coal seam are further analyzed. The test results are basically consistent with the theoretical analysis and numerical simulation. The maximum numerical difference is less than 32%, and the cyclic deformation failure theory of the roadway is verified. That is, the roof of the roadway and the sharp corner of the roof are damaged first. With the increase of load, the two sides of the roadway are divided, and the roof collapses slightly, resulting in the increase of roadway span, the pressure of the two sides is increased, and the damage of the two sides is increased. The stress state at the sharp corner continues to deteriorate. The two sides of the roadway, the two sharp corners of the roof and the sinking of the roof increase the stress, reduce the strength and destroy the vicious circle. Finally, the roadway roof is damaged by asymmetric "Beret" type caving arch, two sides are divided, and the floor heaves slightly.

## References

[1] Sun XM, He MC (2005). Numerical simulation research on coupling support theory of with in soft rock roadway at depth. J. China Univ. Min. Technol..

[2] Wu SQ, Shi PW (1990). The study of rock pressure manifestation in steep seam. J. Xi'an Univ. Min. Technol..

[3] Chen YG, Qian MG (1994). Control of Surrounding Rock in Coal Mines in China.

[4] Fu YK, Wang T, Sun ZY, Zheng JW (2021). Layered control technology and practice of long and short anchor cable in composite soft rock roadway. J. Min Saf. Eng..

[5] Xiong XY, Dai J, Chen XN (2020). Analysis of stress asymmetric distribution law of surrounding rock of roadway in inclined coal seam: A case study of shitanjing no. 2 coal seam. Adv. Civil Eng..

[6] Xiong XY, Dai J (2020). Research on support technology of right angle trapezoidal roadway in gently inclined coal seam. J. China Coal Soc..

[7] Zhong Y. Q. *Theory of Functions of Complex Variables*, 3rd edn. (Education Press, 2004).

[8] Kargar AR, Rahmannejad R, Hajabasi MA (2014). A semi-analytical elastic solution for stress field of lined non-circular tunnels at great depth using complex variable method. Int. J. Solids Struct..

[9] Zhao GP, Yang SL (2015). Analytical solutions for rock stress around square tunnels using complex variable theory. Int. J. Rock Mech. Min. Sci..

[10] Kargar AR, Haghgouei H (2020). An analytical solution for time-dependent stress field of lined circular tunnels using complex potential functions in a stepwise procedure. Appl. Math. Model..

[11] Fang HH, Zhang DL, Fang Q, Wen M (2021). A generalized complex variable method for multiple tunnels at great depth considering the interaction between linings and surrounding rock. Comput. Geotech..

[12] Kong FC, Lu DH, Du XL, Li XQ, Su CC (2021). Analytical solution of stress and displacement for a circular underwater shallow tunnel based on a unified stress function. Ocean Eng..

[13] Liu ZE, Wei YJ (2021). An analytical solution to the stress fields of kinked cracks. J. Mech. Phy. Solids.

[14] Zhao K, Liu CW, Zhang GL (2007). Solution for perimeter stresses of rocks around a rectangular chamber using the complex function of elastic mechanics. J. Min. Saf. Eng..

[15] Li M, Mao XB (2011). Based on the complex variable functions of rectangular roadway rurrounding rock rtress and deformation viscoelastic analysis. Chin. Q. Mech..

[16] Shi GP, Zhu JH, Li BH, Yang JH (2014). Elastic analysis of hole-edge stress of rectangular roadway. Rock Soil Mech..

[17] Xu DQ, Luo XQ (1998). Complex function method for local stability evaluation of underground caverns. Eng. Mech..

[18] Fan GQ, Tang CB (1993). Determination of the mapping function for the exterior domain of non-circular absolutely convergent series. Chin. J. Rock Mech. Eng..

[19] Lv AZ, Wang QW (1995). New method of determination for the mapping function of tunnel with arbitrary boundary using optimization technology. Chin. J. Rock Mech. Eng..

[20] Lv AZ, Zhang LQ (2007). Complex Function Method for Mechanical Analysis of Underground Tunnel.

[21] Zhu DY, Qian QH, Zhou ZS, Xu WH (1999). New method for calculating mapping function of opening with complex shape. Chin. J. Rock Mech. Eng..

[22] Chen L (2020). Study on Fracture Evolution and Instability Mechanism of Large Dip Coal Ceam Roadway Considering the Effect of Mining.

[23] Hu SX (2018). Study on Stability Analysis and Control Technology of Surrounding Rock in Deeply Inclined Coal Seam Roadway.

[24] Zheng ZQ (1992). An approximate method on the conformal mapping from a unit circle to arbitrary curve. Appl. Math. Mech..

[25] Jing W, Guo R, Yang RS, Jing LW, Xue WP (2021). A theoretical analysis of surrounding rock deformation of deep soft rock roadway based on rock rheology and strain softening. J. Min. Saf. Eng..

[26] Shao XX, Chen ZN, Dai YT, Mokhtar M, Xu YJ, Wang CF, Dai ML, Zhu CP, Liu C, Yang FJ, He XY (2017). Research progress of several key problems in digital image correlation method. J. Exp. Mech..

[27] Chai J, Yang YY, Ouyang YB, Zhang DD, Du WG, Li S (2021). Comparison of optical measurement methods for deformation and failure simulation test of overburden in working face. J. China Coal Soc..

[28] Teng YN, Stanier SA, Gourvenec SM (2016). Synchronised multi-scale image analysis of soil deformations. Int. J. Phys. Model. Geotech..

[29] Ghorbani R, Matta F, Sutton MA (2015). Full-field deformation measurement and crack mapping on confined masonry walls using digital image correlation. Exp. Mech..

[30] Pan B, Xie HM, Wang ZY, Qian KM, Wang ZY (2008). Study on subset size selection in digital image correlation for speckle patterns. Opt. Express.

[31] Jia P, Tang CA (2008). Numerical study on failure mechanism of tunnel in jointed rock mass. Tunn. Undergr. Space. Technol..

[32] Xiong XY, Dai J, Ouyang YB, Shen P (2021). Experimental analysis of control technology and deformation failure mechanism of inclined coal seam roadway using non-contact DIC technique. Sci. Rep..

[33] Chai J, Ouyang YB, Liu JX, Zhang DD, Du WG, Lei WL (2021). Experimental study on a new method to forecasting goaf pressure based key strata deformation detected using optic fiber sensors. Opt. Fiber Technol..

[34] Liu Q. Thermal and moisture distribution characteristics of similar materials based on porous media, Doctoral Dissertation. Xi'an University of Science and Technology (2018).

[35] Yoo C, Shin HK (2003). Deformation behaviour of tunnel face reinforced with longitudinal pipes-laboratory and numerical investigation. Tunn. Undergr. Space. Technol..

[36] Hite DJ, Take WA, Bolton MD (2003). Soil deformation measurement using particle image velocimetry (PIV) and photogrammetry. Geotechnique.

[37] Take WA (2015). Thirty-Sixth Canadian Geotechnical Colloquium: advances in visualization of geotechnical processes through digital image correlation. Can. Geotech. J..

[38] Ni P, Wang S, Zhang S, Mei L (2016). Response of heterogeneous slopes to increased surcharge load. Comput. Geotech..

